# Eddy Currents Probe Design for NDT Applications: A Review

**DOI:** 10.3390/s24175819

**Published:** 2024-09-07

**Authors:** Miguel A. Machado

**Affiliations:** 1 UNIDEMI, Department of Mechanical and Industrial Engineering, NOVA School of Science and Technology, Universidade NOVA de Lisboa, 2829-516 Caparica, Portugal; miguel.m@fct.unl.pt; 2 Laboratório Associado de Sistemas Inteligentes, LASI, 4800-058 Guimarães, Portugal

**Keywords:** nondestructive testing, eddy currents testing, eddy currents probes

## Abstract

Eddy current testing (ECT) is a crucial non-destructive testing (NDT) technique extensively used across various industries to detect surface and sub-surface defects in conductive materials. This review explores the latest advancements and methodologies in the design of eddy current probes, emphasizing their application in diverse industrial contexts such as aerospace, automotive, energy, and electronics. It explores the fundamental principles of ECT, examining how eddy currents interact with material defects to provide valuable insights into material integrity. The integration of numerical simulations, particularly through the Finite Element Method (FEM), has emerged as a transformative approach, enabling the precise modeling of electromagnetic interactions and optimizing probe configurations. Innovative probe designs, including multiple coil configurations, have significantly enhanced defect detection capabilities. Despite these advancements, challenges remain, particularly in calibration and sensitivity to environmental conditions. This comprehensive overview highlights the evolving landscape of ECT probe design, aiming to provide researchers and practitioners with a detailed understanding of current trends in this dynamic field.

## 1. Introduction to Eddy Currents

Eddy currents (EC), also known as Foucault currents, represent a fascinating aspect of electromagnetism. These currents arise when a conductive material is subjected to a changing magnetic field. They circulate within the material in closed loops, creating their own magnetic fields in the process. This phenomenon was first observed and described by the renowned French physicist Léon Foucault in 1855 [1].

**Figure 1:**
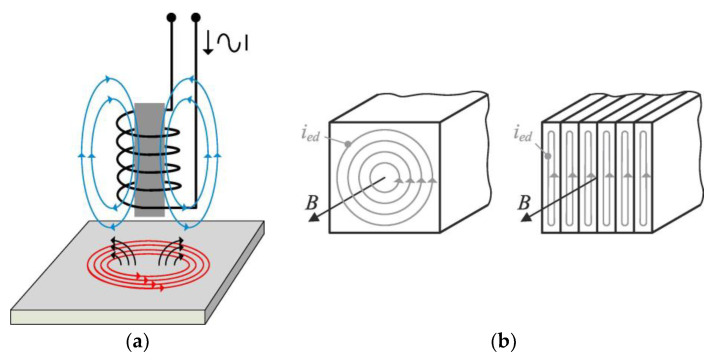
Illustration of eddy currents: (**a**) visualization of eddy currents (in red) and their associated magnetic fields [21]; (**b**) eddy currents induced by magnetic field B in both solid and laminated transformer cores [8].

Foucault’s discovery of eddy currents marked a significant milestone in the understanding of electromagnetic interactions. He demonstrated that when a conductor, such as a metal plate, is exposed to a varying magnetic field, electric currents are induced within the material. These currents arise due to the electromagnetic induction principle, whereby a changing magnetic field induces an electromotive force and subsequently generates a current flow. The interaction between the induced currents and the original magnetic field results in the formation of secondary magnetic fields, which oppose the change in the applied magnetic field [2,3]. Figure 1a contains a graphical representation of eddy currents being generated on a metal plate through a bobbin coil and the inherent magnetic fields.

Eddy currents were initially perceived as unwanted since they can pose challenges in various contexts, particularly in electrical systems and transformers, where they contribute to energy losses through heat dissipation. Therefore, in Brazil, the term coined for eddy currents is “parasite currents” (literal translation) since their presence is not always desirable [4]. In electrical transformers and other electromagnetic devices, eddy currents result in energy losses due to resistive heating in the conductive materials [1,5]. These losses can lead to decreased efficiency and increased operating costs, making it imperative to minimize their impact. Engineers and researchers have devised various strategies to mitigate eddy current losses, including the use of laminated or segmented core materials (Figure 1b), which effectively disrupt the continuous flow of eddy currents, reducing energy losses and improving overall efficiency [6,7,8]. Beyond electrical systems, eddy currents can also complicate the design and operation of high-speed rotating machinery, such as generators, motors, and turbines [9,10,11]. In such applications, precise modeling and simulation techniques are employed to predict and optimize the behavior of eddy currents, ensuring the reliability and performance of critical systems [12,13,14]. In industrial processes involving magnetic materials, eddy currents can also interfere with desired outcomes, causing distortions in magnetic fields and affecting material properties. For example, in magnetic levitation (maglev) trains, eddy currents induced in the metallic rails can create drag forces, reducing the efficiency and speed [15,16,17]. Similarly, in metal-forming processes, such as induction heating and welding, eddy currents must be carefully managed to achieve desired heating or joining effects without inducing unwanted distortions or defects [18,19,20].

Despite these challenges, eddy currents offer immense potential for beneficial applications across various industries, particularly in the realms of non-destructive testing (NDT) and non-destructive evaluation (NDE), including materials’ characterization and defect detection. NDT encompasses a range of inspection methods used to evaluate the integrity, properties, and quality of materials, components, and structures without causing damage [22]. NDT techniques are essential in various industries to ensure the safety, reliability, and performance of critical assets. Common NDT techniques include ultrasonic testing [23,24], radiographic testing [25], magnetic particle testing, dye penetrant testing [26], thermography [27,28,29], terahertz inspection [30], and eddy currents [31]. Furthermore, NDT techniques often intersect with Structural Health Monitoring (SHM), a field focused on continuously monitoring the condition of structures to detect damage or degradation. SHM integrates NDT methods with sensors [32], data analysis algorithms [33,34], and structural models to provide real-time information on structural health [35,36], enabling proactive maintenance and minimizing the risk of catastrophic failure [37,38,39,40,41]. This synergy between NDT and SHM enhances asset management practices, ensuring the long-term reliability and safety of critical infrastructure.

Eddy Current Testing (ECT) leverages the changes in the magnetic field induced by eddy currents when they encounter heterogeneities within the material. As with any electrical current, eddy currents tend to follow the path of least resistance. When they encounter heterogeneities such as superficial cracks, voids, or material inconsistencies, the paths of these eddy currents are inevitably changed. These disruptions in the eddy current flow result in deviations in the secondary magnetic field, which can be precisely measured and analyzed [42]. By interpreting these deviations, ECT technicians can identify and evaluate defects within the material, providing valuable insights into its structural integrity and quality.

This capability makes eddy current testing invaluable in industries such as aerospace [43,44], automotive, energy [45,46], manufacturing [47,48], and material science [49], where the integrity and reliability of critical components are paramount.

### Industrial Applications of ECT

The versatility of ECT extends far beyond defect detection, encompassing a wide range of applications tailored to meet the specific requirements of diverse industries. This NDT technique offers unparalleled flexibility in terms of the inspection speed [50], temperature tolerance [51,52,53], and the ability to perform contact or non-contact inspections [54], making it an indispensable tool in various sectors.

In the automotive industry, eddy current testing serves as a cornerstone of quality control processes, particularly during the production of critical components such as engine parts and chassis welds. ECT techniques are employed to inspect these components for surface defects, cracks, or material inconsistencies that could compromise performance or safety [55]. With its ability to rapidly assess large volumes of parts, eddy current testing helps ensure that automotive components meet stringent quality standards and regulatory requirements. Moreover, eddy current testing is utilized for sorting and classifying automotive components based on material properties such as conductivity or hardness. By accurately categorizing parts according to their quality attributes, manufacturers can optimize production efficiency and minimize waste, ultimately enhancing cost-effectiveness and competitiveness in the automotive market [56].

In the aerospace sector, where safety and reliability are paramount, eddy current testing plays a critical role in inspecting aircraft structures, engine components, and composite materials for hidden defects or structural anomalies [57]. ECT techniques are employed to detect surface cracks, corrosion, delamination, or disbonds that may compromise the structural integrity of aerospace components [58].

Eddy current testing is particularly well-suited for inspecting complex geometries and hard-to-reach areas, making it an indispensable tool for maintaining the airworthiness of aircraft and ensuring compliance with rigorous safety standards. Whether conducting routine maintenance inspections or evaluating newly manufactured components, aerospace engineers rely on eddy current testing to identify and mitigate potential risks associated with material degradation or manufacturing defects [59].

In the energy sector, eddy current testing plays a crucial role in inspecting critical components such as pipelines, pressure vessels, and heat exchangers used in oil and gas production, refining, and distribution [60,61]. ECT techniques are employed to detect corrosion, erosion, and other defects that could compromise the structural integrity and safety of these assets. By identifying potential issues early, ECT helps to prevent costly failures, minimize downtime, and ensure the reliable operation of energy infrastructure [62,63].

In the electronics industry, eddy current testing is used for inspecting printed circuit boards (PCBs), electronic components, and assemblies for defects such as cracks, delamination, and solder joint integrity issues [64]. ECT techniques enable the rapid and non-destructive assessment of electronic devices, ensuring compliance with quality standards and reliability requirements. Additionally, eddy current testing is employed in the semiconductor industry for wafer inspection, detecting defects such as cracks, voids, and metal contamination that could affect the device performance and yield [65].

In material science research and development, eddy current testing is employed for characterizing material properties, evaluating material microstructures, and studying electromagnetic phenomena. ECT techniques are used to measure parameters such as electrical conductivity [66], magnetic permeability, and material thickness, providing valuable insights into material behavior and performance [67,68,69]. Researchers leverage eddy current testing to study the effects of heat treatment, alloy composition, and processing techniques on material properties, enabling the development of advanced materials for various applications, including aerospace, automotive, and renewable energy technologies [70].

Despite being initially perceived as unwanted due to their potential to cause energy losses and operational challenges, eddy currents have evolved into a pivotal aspect of modern engineering and technology. By understanding and harnessing the electromagnetic interactions underlying eddy currents, researchers and engineers have unlocked a plethora of beneficial applications, particularly in the realm of NDT. This article seeks to shed light on both the challenges posed by eddy currents and their immense potential for innovation and advancement. Through a thorough exploration of eddy current principles, industrial applications, and ongoing research efforts, this review aims to provide readers with a comprehensive understanding of this fascinating phenomenon and its relevance in today’s engineering landscape. Specifically, we will explore different inspection scenarios, including various industries and materials, where eddy current techniques have been applied to detect defects, characterize materials, and ensure the integrity of critical components.

## 2. Fundamentals of Eddy Current Testing

**Figure 2:**
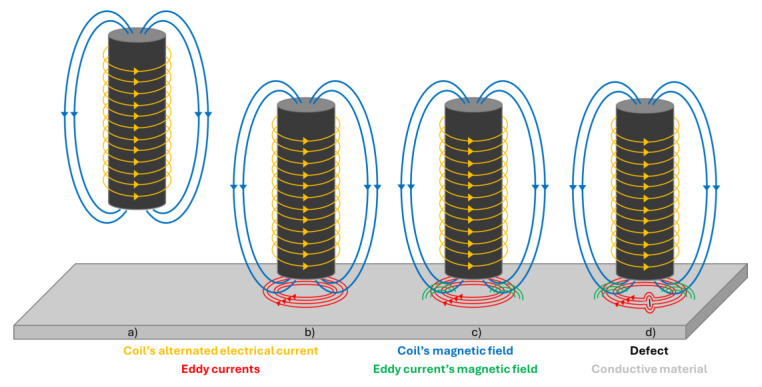
Illustration of ECT principles: (**a**) Coil with alternating current in air, or with a very high lift-off, generating an alternating magnetic field around the coil. In this situation, the magnetic field does not effectively reach the conductive material, resulting in no EC being induced. (**b**) When the coil is over a conductive material, the alternating magnetic field from the coil generates EC in the material. (**c**) Eddy currents generate their own magnetic field (secondary magnetic field) which opposes the original magnetic field from the coil. (**d**) In the presence of a defect in the material, the ECs divert around the defect, causing a disturbance in their normal flow and the secondary magnetic field, indicating the presence and location of the defect.

Eddy Current Testing (ECT) relies on the principles of electromagnetic induction to assess the integrity of materials and components. When an alternating current flows through a coil in the ECT probe, it generates oscillating magnetic fields near the surface of the material being inspected (Figure 2a). These alternating magnetic fields induce eddy currents (EC) to circulate within the conductive material, as seen in Figure 2b. The key principle behind ECT lies in the interaction between these induced eddy currents and the material under inspection. As eddy currents circulate within the material, they generate their own magnetic fields (Figure 2c). The initial distribution of these eddy currents is primarily influenced by the material’s electrical conductivity, magnetic permeability, and geometrical properties. In turn, the behavior and distribution of the resulting secondary magnetic fields are directly shaped by the eddy current pattern, which is modulated by these material properties. Therefore, the material’s properties influence the secondary magnetic fields indirectly through their control over the eddy current distribution. When eddy currents encounter defects such as cracks, voids, or material inconsistencies, their paths are deviated, as depicted in Figure 2d. This disruption in the eddy current flow leads to changes in the secondary magnetic field surrounding the material. By measuring and analyzing these changes, ECT technicians can detect defects within the material. ECT boasts several advantages, making it a popular choice for non-destructive testing applications. Firstly, ECT is highly sensitive to surface and near-surface defects in conductive materials, allowing for the detection of flaws such as cracks, voids, and material inconsistencies. This sensitivity makes ECT particularly useful in industries where component reliability is critical, such as aerospace, automotive, and manufacturing.

Another advantage of ECT is its versatility. It can be applied to a wide range of materials, including metals, alloys, and non-ferrous materials, making it suitable for various industrial applications. Additionally, ECT is relatively fast and efficient, enabling the rapid inspection of components without the need for extensive preparation or dismantling. The impact of velocity on eddy currents has been studied in straightforwardly shaped specimens, such as bars, tubes, and wires, moving at high speeds. Simulations have explored very low frequencies (e.g., 40 Hz) and velocities of up to 1000 m/s [71]. Moreover, ECT is non-destructive, meaning it does not alter or damage the inspected material during testing. This makes it an ideal choice for quality control and assurance, allowing manufacturers to assess component integrity without compromising their structural or functional properties.

Despite its many advantages, ECT also has some limitations and challenges. One drawback is its sensitivity to surface conditions such as roughness and cleanliness. Surface irregularities can affect the accuracy of ECT inspections by altering the lift-off distance between the probe and the material surface, leading to inaccurate results [72,73]. Additionally, ECT is primarily limited to conductive materials, as it relies on the induction of eddy currents to detect defects. This restricts its applicability in industries where non-conductive materials are prevalent.

A significant challenge of ECT is its limited penetration depth. Eddy currents generated by the probe are primarily confined to the surface or near-surface regions of the material, a phenomenon known as the skin effect. This effect causes the density of eddy currents to decrease exponentially with depth according to Equation (1), making ECT less effective for detecting deep defects. Equation (1) shows how the current density, *I_x_* [A∙m^−2^], varies with depth, *x* [m], where *I*_0_ [A∙m^−2^] is the current density at the surface, *f* [s^−1^] excitation frequency, *μ* [H∙m^−1^] is the magnetic permeability (*μ* = *μ*_0_ · *μ_r_*), and *σ* [S/m] is the electrical conductivity.

(1) $$I_{x}=I_{0}\cdot e^{-x\sqrt{\pi \cdot \;f\;\cdot \;\mu \;\cdot \;\sigma }}$$

The maximum EC penetration depth, often referred to as the standard penetration depth, *δ* [m], is defined as the depth at which the current density (*I_x_*) is e^−1^ (approximately 37%) of the current density at the material surface (*I*_0_), assuming a plane wave magnetic field. This calculation assumes an idealized plane wave magnetic field, which is a condition rarely achieved in conventional coil probe setups. Nevertheless, this approximation remains useful for estimating the penetration depth in ECT applications. The standard penetration depth is calculated using Equation (2) where *f* [s^−1^] is the excitation frequency, *μ* [H ∙ m^−1^] is the magnetic permeability (*μ* = *μ*_0_ ∙ *μ_r_*), and *σ* [S/m] is the electrical conductivity [74].

(2) $$\delta_{\left(f,\mu ,\sigma \right)}=\frac{1}{\sqrt{\pi \cdot \;f\;\cdot \;\mu \;\cdot \;\sigma }}$$

In practice, EC can penetrate deeper than this standard depth of penetration. Depending on the probe geometry, it is possible to achieve current densities slightly higher than 37% at greater depths. This extended penetration capability has been experimentally validated in certain EC probes [75].

**Figure 3:**
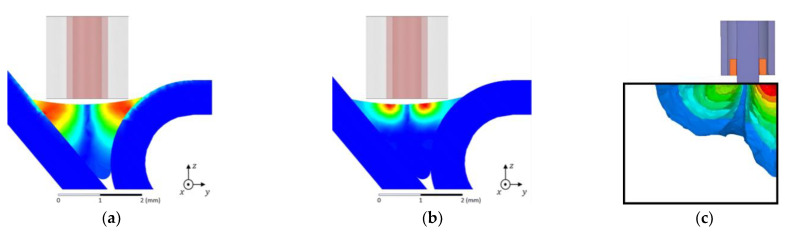
Numerical simulation of EC density distribution: (**a**) low frequency which allows an increased penetration depth; (**b**) high frequency which causes a pronounced skin effect; and (**c**) distortion in the EC flow by the edge (adapted from [55,77]).

The skin effect’s intensity depends on the frequency of the excitation current and the material’s properties. Higher frequencies result in shallower penetration depths, and materials with high conductivity and magnetic permeability exhibit more pronounced skin effects, further reducing the penetration depth. Several strategies can be employed to address this challenge. Utilizing lower frequencies can increase the penetration depth (Figure 3a,b), although this may reduce the resolution and sensitivity for detecting smaller, surface-level defects. Pulsed Eddy Current (PEC) Testing, which uses transient or pulsed signals instead of continuous sinusoidal waves, can provide information about deeper layers by analyzing the decay of induced eddy currents over time.

The edge effect in ECT is a phenomenon that occurs when the eddy current probe is placed near the edge or boundary of a conductive material [76,77]. This effect is characterized by a distortion in the eddy current flow and the resulting magnetic field, leading to variations in the signal detected by the probe (Figure 3c). The edge effect can significantly impact the accuracy of defect detection and characterization, as it introduces noise and false indications that can be mistaken for flaws. This is particularly problematic in components with complex geometries or small surface areas, where edges are more prevalent [78].

In eddy current testing, the excitation current plays a crucial role in determining the performance and effectiveness of the eddy current probe. The key parameters—magnitude, frequency, and phase—each significantly impact the probe’s capabilities. The magnitude of the excitation current affects the overall signal strength and sensitivity of the probe. A higher magnitude enhances the ability to detect smaller or subtler defects by increasing the induced eddy currents in the material [79]. However, if the magnitude is too high, it can lead to signal saturation and potential overheating, which might compromise the measurement accuracy. Therefore, it is important to find an optimal balance to achieve both high sensitivity and reliable signal interpretation.

The frequency of the excitation current directly influences the depth of penetration and the resolution of the probe. Higher frequencies result in shallower penetration, making the probe more adept at detecting surface and near-surface defects, but less effective for deeper flaws [80]. Conversely, lower frequencies allow for deeper penetration, enabling the detection of defects at greater depths, but potentially reducing the sensitivity to finer surface details. Thus, the choice of frequency must align with the specific requirements of the inspection task and the properties of the material being tested.

The phase of the excitation current also plays a critical role in eddy current testing by affecting the phase relationship between the induced eddy currents and the detected signal. Adjustments to the phase can enhance the probe’s sensitivity to specific types of defects or material variations by altering the phase of the detected signal relative to the excitation current [81]. This capability is especially useful in complex inspection scenarios that require precise defect characterization.

Understanding and optimizing the magnitude, frequency, and phase of the excitation current are essential for maximizing the performance of eddy current probes. By carefully tuning these parameters, it is possible to tailor the probe’s functionality to meet specific inspection needs, thereby improving the accuracy and reliability of the testing process.

Building upon the fundamental principles of the excitation current, ECT has evolved through several advanced techniques. These advancements include single-frequency [82], which provides a basic approach to defect detection, multiple-frequency [55,83], which enhances sensitivity across various depths, swept-frequency ECT [84,85], which allows for a dynamic inspection across a range of frequencies, and pulsed or transient ECT [86,87], which offers improved resolution and defect characterization by analyzing the response to short-duration pulses. Each of these methods leverages the principles of an excitation current in different ways to address diverse inspection challenges. Initially, ECT primarily employed single-frequency methods, which remain prevalent for detecting surface and near-surface cracks. This basic technique typically uses a sinusoidal current, with frequencies ranging from hundreds of Hz to several MHz, chosen based on the material and defect depth [88]. Higher frequencies are used for surface defects, while lower frequencies are better for deeper flaws. Single-frequency systems generally include an oscillator, an excitation coil, one or more sense coils, signal processing components, and an impedance plane display for data interpretation. The coils can be integrated into a single unit or separated, with designs tailored to specific applications.

However, single-frequency ECT has limitations in identifying multiple test conditions. To address this, multi-frequency techniques have been introduced, providing enhanced results, especially for ferromagnetic materials [89]. These methods offer a better analysis of complex flaws and can distinguish between defects and variations in conductivity, permeability, geometry, and probe lift-off by subtracting characteristic signals from these variations [90].

Pulsed Eddy Current (PEC) technology, with its broad frequency spectrum, can assess various parameters such as the defect size, location, and probe lift-off. PEC systems can also measure thickness at large lift-off distances (up to 100 mm), making them useful for detecting corrosion under insulation [91]. PEC techniques show promise for materials with high conductivity, such as copper, where single-frequency methods often fail [92]. This approach enables the simultaneous detection of both near-surface and deeper flaws without changing the probe or frequency. The development of PEC has been supported by advances in computing and signal processing, though its adoption is limited due to the nascent stage of transient response interpretation [93]. PEC, also known as a transient eddy current, involves driving a large, pulsed current through the excitation coil, creating transient eddy currents in the specimen. The resulting signal, rich in low-frequency components, provides insights into subsurface defects. Longer return times for deeper signal components facilitate quantitative NDT, as each component reflects different depths.

### 2.1. ECT Probe Configurations and Operation Modes

ECT employs a range of probe configurations and operational modes tailored to specific inspection requirements. This section covers single coil probes, valued for their simplicity, and extends to multiple coil probes, which include differential, reflection, and bridge modes, each enhancing defect detection capabilities. These probes are integral in inspecting diverse materials and structures, demonstrating the versatility and adaptability of ECT technologies.

#### 2.1.1. Single-Coil Probes

**Figure 4:**
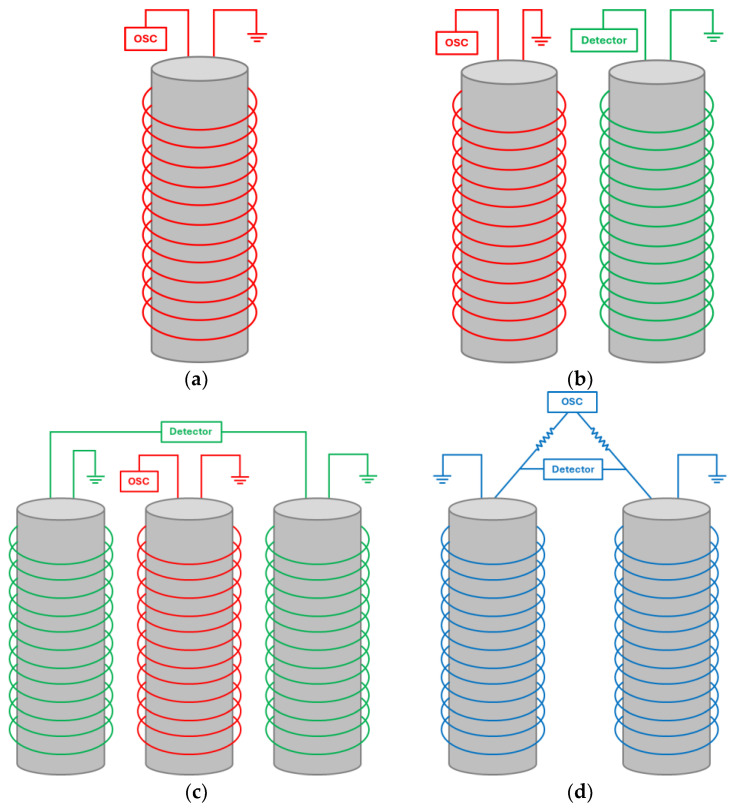
Schematic diagram of ECT probe types: (**a**) single coil—absolute probe; (**b**) reflection probe with one driver coil and one pick-up coil, generating an absolute-type output; (**c**) reflection probe with one driver coil and two pick-up coils with differential measurement between the pick-up coils; (**d**) bridge differential probe with two coils both working as driver and pick-up coils in which the result obtained is differential.

Single-coil probes, also known as absolute probes, use a single coil for both excitation and sensing (Figure 4a). This coil generates the eddy currents in the test material and the same coil then measures the response of these currents, providing an absolute signal. In the absolute mode, the probe directly measures the total signal, making it suitable for applications where a baseline or reference measurement is not required. This design is simple and effective for detecting surface and near-surface defects. However, it is less sensitive to sub-surface defects and more affected by lift-off variations and surface roughness.

#### 2.1.2. Multiple-Coil Probes

Multiple-coil probes incorporate several coils configured in various ways to enhance defect detection. These probes provide increased flexibility, improved spatial resolution, and greater sensitivity, making them suitable for more complex inspection tasks. They offer better depth penetration and defect characterization, but are more complex in design and implementation, requiring advanced signal processing.

#### 2.1.3. Multiple Coil Probes—Differential

Differential probes are used to measure the differential response between two points on the material surface, helping to improve inspection accuracy and sensitivity. By measuring the difference in response between two points on the material surface, differential probes enhance the sensitivity to subtle variations in the eddy current behavior. This heightened sensitivity allows for the detection of smaller defects or inconsistencies that may not be discernible with single-coil probes. They also help to minimize the impact of environmental factors such as temperature variations or electromagnetic interference. This can result in more reliable and consistent inspection results, particularly in challenging operating environments. Differential probes can operate in two configurations: differential excitation and differential pick-up. In differential excitation, two coils are driven by opposing currents, which accentuates local variations caused by defects while cancelling out uniform background signals. In differential pick-up, the response difference between two closely spaced sensing coils is measured, enhancing the detection of small, localized defects. These probes can be used in conjunction with both reflection and absolute mode configurations, offering versatility in inspection techniques. This versatility allows one to tailor the approach based on the specific requirements of the inspection, ensuring an optimal performance across a wide range of materials and geometries.

#### 2.1.4. Multiple-Coil Probes—Reflection Probes

Reflection probes, or transmission probes, use separate excitation and detection coils. The driver coil induces eddy currents in the material, while one or more pick-up coils measure the response. This separation allows for the clearer detection of the induced eddy currents and their response, leading to improved signal-to-noise ratios and enhanced sensitivity in defect detection. It also provides greater flexibility in probe design [94]. These probes can operate in the absolute mode, measuring each pick-up coil’s response independently, as depicted in Figure 4b, or in the differential mode, comparing responses between multiple pick-up coils to detect anomalies, as shown in Figure 4c. The ability to switch between these modes offers adaptability in various inspection scenarios. In the differential mode, the probe becomes more sensitive to local variations and can better detect small defects by comparing the differences between signals from adjacent pick-up coils. Keeping the excitation and sensing coils separate minimizes the signal interference between them. The positioning and orientation of each coil can be optimized to maximize the signal strength and accuracy, adapting the probe to specific inspection requirements and geometries. Ona et al. found that both the coil gap and lift-off influence probe sensitivity which can be optimized through the selection of a driver-pick-up coil gap and lift-off [95].

#### 2.1.5. Multiple-Coil Probes—Bridge Probes

Bridge probes feature a configuration where two coils act as both the driver and pick-up coils, typically measured in the differential mode, as seen in Figure 4d. This setup forms a bridge circuit, allowing the probe to detect changes in the magnetic field caused by defects by measuring the imbalance between the coils. Bridge probes are highly sensitive to small changes in material properties, making them particularly effective for detecting minor defects or subtle variations in conductivity. Additionally, the differential configuration helps to effectively minimize noise and interference, enhancing signal clarity by cancelling out common-mode signals such as background noise or uniform material variations.

This design can operate in both differential excitation and differential pick-up modes, further enhancing its adaptability based on specific inspection needs. In the differential excitation mode, two coils are driven by alternating currents in opposite directions. This setup creates opposing magnetic fields that cancel each other out in areas without defects, producing minimal output. When a defect is present, the symmetry is disrupted, causing a detectable change in the output signal. This configuration enhances the sensitivity to local defects while minimizing background noise. In the differential pick-up mode, the probe uses closely spaced sensing coils to compare the response at two adjacent points on the material. In areas without defects, the signals from both coils are nearly identical and cancel each other out. However, when a defect is encountered, the imbalance between the signals becomes evident, allowing for defect detection. This mode is particularly useful in environments with varying material properties or geometries, where it is crucial to isolate small defects from broader variations. Bridge probes can switch between these differential modes based on inspection needs, offering flexibility in a wide range of testing scenarios. However, the complexity of the calibration and precise coil alignment remain critical challenges, as any misalignment can lead to incorrect readings or reduced sensitivity.

### 2.2. ECT Probe Technologies and Sensing Solutions

The evolution of ECT probes includes a transition from traditional wound probes to innovative planar probes, such as those utilizing printed circuit board (PCB) technology. Array probes, designed for efficient large-area coverage, are also highlighted, along with advanced sensor types like Hall effect sensors, Giant Magnetoresistance (GMR) sensors, and Superconducting Quantum Interference Devices (SQUIDs). This section delves into these diverse designs and sensors, showcasing advancements in ECT that enhance the precision, sensitivity, and application scope.

#### 2.2.1. Wound Probes

**Figure 5:**
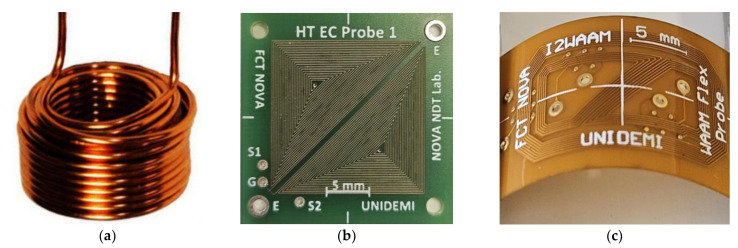
ECT probe manufacturing technologies: (**a**) helicoidal cylindrical coil with air core; (**b**) planar spiral rigid PCB probe; and (**c**) flexible PCB probe.

Traditional wound probes embody a time-tested approach to eddy current probe design. These probes typically feature a coil wound around a core material, such as ferrite or laminated iron, forming the primary component of the probe assembly. The winding process involves wrapping conducting wire around the core in a precise configuration, ensuring optimal electromagnetic coupling and sensitivity to variations in the material under inspection. Alternatively, some probes may utilize air as the core material (no core), which simplifies the design and eliminates the potential magnetic interference from core materials, as depicted in Figure 5a. The design may vary based on factors such as the probe size, frequency range, and intended application, with larger probes often featuring multiple windings to enhance the sensitivity and signal strength. Additionally, the choice of the core material plays a crucial role in determining the probe’s performance characteristics, with different materials offering varying degrees of magnetic permeability and electrical conductivity.

Ferrite shields, in particular, are often employed in these probes to enhance the performance by concentrating the magnetic field within the core and reduce the external electromagnetic interference. This improves the signal-to-noise ratio and overall sensitivity of the probe. By focusing the magnetic field, ferrite shields also help to improve the spatial resolution of the probe, making it more effective at detecting small defects. Despite advancements in probe technology, traditional wound probes remain a cornerstone in NDT applications, valued for their reliability, simplicity, and effectiveness in detecting defects and anomalies in a wide range of materials and geometries.

#### 2.2.2. Planar Probes—Print Circuit Board (PCB)

Planar ECT probes, such as those based on printed circuit board (PCB) technology, represent a modern and innovative approach to eddy current testing (Figure 5b). By leveraging the principles of PCB technology, these probes offer compact, lightweight, and highly customizable inspection tools [96]. Unlike traditional coil winding processes, PCB probes use etched conductive traces on a PCB substrate to form the primary sensing element [97]. This design allows for precise control over the coil geometry, spacing, and configuration, enhancing the sensitivity and signal-to-noise ratio. Additionally, the scalability of PCB manufacturing facilitates rapid prototyping and mass production tailored to specific inspection needs [98]. The integration of advanced electronics and signal processing capabilities directly onto the PCB substrate enables real-time data acquisition, analysis, and communication. This integration supports seamless operation with automated inspection systems and data logging software. Furthermore, PCB probes can be customized for various configurations, including single-coil, multi-coil arrays, and multiplexed designs, catering to diverse inspection scenarios and material types. PCB probes are also versatile and adaptable to emerging technologies and industry trends [99]. Advancements in materials science, miniaturization, and manufacturing processes continue to improve the performance, reliability, and functionality of these probes in NDT applications. Industries such as aerospace, automotive, manufacturing, and infrastructure increasingly rely on PCB probes for efficient, cost-effective, and high-performance solutions for defect detection and material characterization [100].

The PCB probes can also be manufactured using flexible substrates, offering additional advantages due to the inherent properties of these materials. Flexible PCB probes are composed of thin, flexible materials that allow them to conform to curved or irregular surfaces with ease, as seen in Figure 5c. This capability makes them particularly suitable for inspecting components with complex geometries, such as turbine blades, pipes, and curved surfaces, where traditional rigid probes may not provide optimal contact or coverage. These flexible PCB probes maintain high sensitivity and reliability by integrating advanced electronics onto flexible substrates. They offer improved maneuverability and contact with the inspected surface, ensuring the effective inspection of components in aerospace, automotive, and manufacturing industries where flexibility, adaptability, and precision are critical. However, challenges such as thermal management and mechanical durability must be addressed in the design of flexible PCB probes. Flexible PCB materials typically have a lower thermal resistance compared to rigid PCBs, which may limit their operation in high-temperature environments without adequate thermal mitigation strategies. Ensuring robust mechanical reliability and consistent electrical performance over time are critical considerations in deploying flexible PCB probes for long-term NDT applications. Despite these challenges, the adaptability, lightweight nature, and customization capabilities of flexible PCB probes make them increasingly valuable in modern NDT practices. Their ability to efficiently detect defects and characterize materials with high precision and flexibility positions them as essential tools in enhancing quality assurance and asset integrity management across diverse industrial sectors.

#### 2.2.3. Array ECT Probes

**Figure 6:**
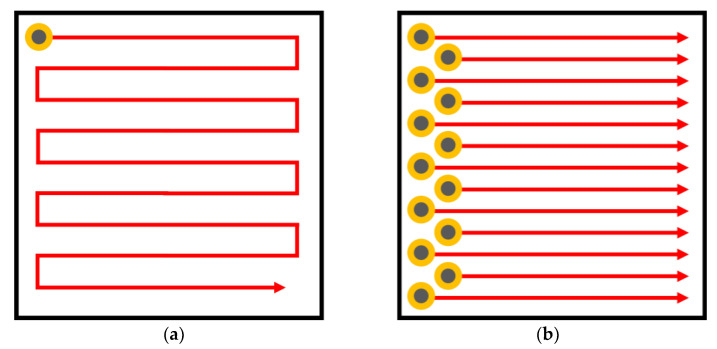
Schematic diagram of ECT scans with (**a**) single coil and (**b**) ECA probe covering the same area.

Eddy current arrays (ECA) are advanced configurations of eddy current testing probes designed to enhance inspection capabilities and efficiency. Unlike traditional single-coil probes, ECA systems use multiple coils arranged in a specific pattern, allowing for simultaneous data acquisition over a larger area, as depicted in Figure 6. This arrangement significantly reduces the inspection time and increases the probability of defect detection. The primary advantage of ECA is its ability to provide detailed and comprehensive coverage, which is particularly useful for inspecting complex geometries and large surfaces. ECAs can detect both surface and subsurface defects with high precision, and their ability to produce C-scan images enables better visualization of defects.

ECA systems employ multiplexing techniques to manage the simultaneous operation of multiple coils, which helps in minimizing interference and optimizing data quality. This multiplexing capability allows for faster area coverage and the generation of high-resolution C-scans and B-scans, providing valuable insights into the material’s condition [101].

The design of ECA probes can vary, with both three-dimensional and planar configurations available. The coils in these arrays can be either traditional wound coils or PCB coils, each offering distinct advantages. Wound coils typically provide higher sensitivity and deeper penetration, making them suitable for detecting subsurface defects. In contrast, PCB coils are more versatile and can be precisely manufactured to fit specific inspection requirements, especially for planar surfaces [102].

Despite their numerous advantages, ECA systems also have some disadvantages. They are generally more expensive than traditional single-coil probes due to their complexity and the need for sophisticated data acquisition and processing systems. Additionally, the increased complexity of ECA systems can lead to a more challenging calibration and interpretation of results, requiring highly skilled operators. Another challenge is that the resolution of ECA may be limited by the spacing of the coils in the array; closely spaced defects may be difficult to distinguish [103].

ECA technology addresses several problems inherent in traditional eddy current testing, such as limited coverage and the need for multiple scans, by providing faster, more reliable, and detailed inspections, making it an invaluable tool in various industries.

#### 2.2.4. Other Sensors Used in ECT

In addition to traditional coil-based ECT, several advanced sensor technologies enhance ECT capabilities and address specific inspection challenges such as Hall effect sensors, Giant Magnetoresistance (GMR) sensors, and SQUID (Superconducting Quantum Interference Device) sensors. These sensors can be integrated into planar or three-dimensional coil designs, allowing for versatile application in ECT and enabling detailed magnetic field mapping and high-precision flaw detection in various materials.

##### Hall Effect Sensors

Hall sensors are highly sensitive and capable of detecting minute changes in magnetic fields, which is crucial for identifying small or subsurface defects. This precision makes them versatile, allowing integration into both planar and three-dimensional coil designs. This versatility ensures that Hall effect sensors can be used across various applications, from inspecting flat surfaces to complex geometries. In real-world applications, Hall sensors are instrumental across several industries. In pipeline inspection, for example, Hall sensors detect corrosion and cracks, allowing for early defect detection and preventing leaks and potential failures [104,105,106]. The aerospace industry utilizes Hall sensors for the safety-critical inspection of aircraft components, identifying cracks and corrosion in metal parts to ensure structural integrity [107,108]. Similarly, the automotive sector benefits from Hall sensors in inspecting welds and metal components, contributing to vehicle safety and reliability. In power generation, Hall sensors help detect stress corrosion cracking in turbines and other critical infrastructure, ensuring an uninterrupted power supply and safety. Several case studies illustrate the effectiveness of Hall effect sensors. In Pipeline Inspection Gauges (PIGs), Hall sensors detect very small changes in the magnetic field caused by defects in pipeline walls [109,110,111,112]. In aircraft wheel inspections, Hall sensors identify subsurface cracks, critical for the maintenance and safety of aircraft. Electric vehicle battery inspections also benefit from Hall sensors, ensuring the safety and longevity of battery packs by detecting defects in the battery casing. Integrating Hall effect sensors into ECT provides a higher level of defect detection and analysis, enhancing the safety and reliability of critical components. Their sensitivity, precision, and versatility make Hall effect sensors a valuable addition to traditional ECT methods [113].

##### Giant Magnetoresistance Sensors

Giant Magnetoresistance (GMR) sensors have transformed ECT by offering high sensitivity and spatial resolution for detecting magnetic fields associated with eddy currents. GMR sensors excel in identifying minute changes in magnetic fields, making them particularly effective for detecting small and deep-seated defects that traditional ECT methods might overlook [114,115,116]. One of the significant advantages of GMR sensors is their ability to provide high-resolution measurements. This allows for the more detailed imaging and mapping of defects within materials. GMR sensors can be incorporated into both planar and three-dimensional configurations, enhancing their applicability in inspecting various geometries and complex structures. Their compact size and high sensitivity enable them to be used in applications where space is limited and precise defect detection is critical [117]. Several case studies highlight the effectiveness of GMR sensors in ECT. For example, in aerospace applications, GMR sensors have been used to detect corrosion under insulation (CUI) in aircraft fuselage panels, providing early detection and preventing potential failures [118,119,120]. In the automotive industry, GMR sensors have been utilized to inspect spot welds in vehicle bodies, ensuring strong and reliable joints [121]. In power generation, GMR sensors have been applied to monitor turbine blades for cracks and other defects, ensuring the reliability and safety of power plants [122,123]. Integrating GMR sensors into ECT systems significantly enhances the ability to detect and analyze defects, providing high-resolution and precise measurements. Their sensitivity, compact size, and versatility make GMR sensors a valuable addition to traditional ECT methods, improving the reliability and safety of critical components across various industries [124,125,126].

##### Superconducting Quantum Interference Devices

Superconducting Quantum Interference Devices (SQUIDs) represent an advanced technology in ECT, offering unparalleled sensitivity to magnetic fields. SQUIDs operate at extremely low temperatures and utilize superconducting loops containing Josephson junctions to detect even the faintest magnetic signals generated by eddy currents. This extreme sensitivity allows SQUIDs to detect very small defects and to measure magnetic fields generated deeper within materials compared to traditional ECT sensors [127,128]. Unlike conventional probes, SQUIDs do not transmit magnetic fields, but instead detect the weak magnetic responses from eddy currents at greater depths. The use of SQUIDs in ECT is particularly beneficial in applications requiring the detection of subtle flaws or inclusions within conductive materials. For example, in the aerospace industry, SQUIDs have been employed to identify micro-cracks and other minute defects in critical components like turbine blades and aircraft fuselages. Their ability to detect these defects at an early stage enhances the safety and longevity of aerospace components [129,130]. SQUIDs have also been applied in the energy sector, particularly in the inspection of nuclear reactors and other critical infrastructure [131,132]. Their capability to detect tiny cracks and other defects in reactor components ensures the continued safe operation of these facilities, preventing potential failures and reducing maintenance costs. While the implementation of SQUIDs in ECT presents significant advantages, including high sensitivity and the ability to detect deep-seated defects, there are also challenges [133]. The requirement for extremely low operating temperatures and the complexity of SQUID technology can make these systems costly and difficult to maintain [134]. However, ongoing advancements in cryogenics and SQUID technology are helping to mitigate these challenges, making SQUID-based ECT an increasingly viable option for a wide range of applications.

## 3. Eddy Current Probes’ Designs and Solutions

**Figure 7:**
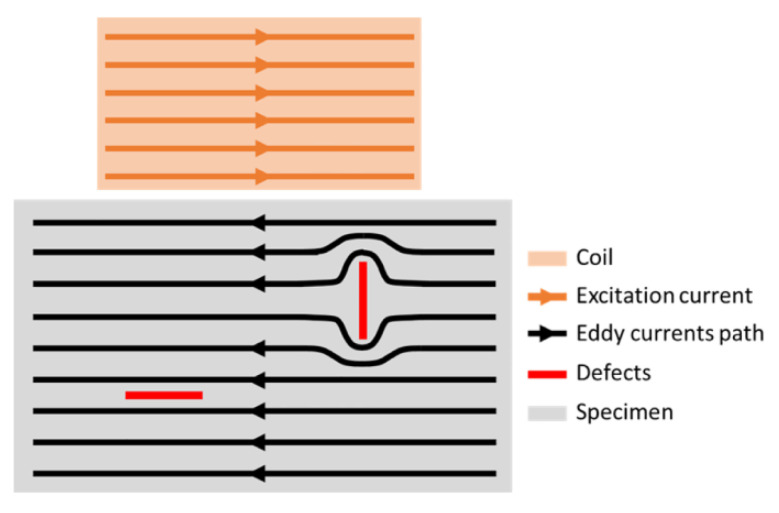
Schematic illustrating the impact of defects on EC flow: A defect positioned transversely to the EC direction can cause a significant deviation in the EC path, which can be detected by the probe due to the altered electromagnetic response. In contrast, a defect aligned with the EC direction causes minimal or no disruption to the EC flow, making it more challenging to detect [135].

Eddy current testing (ECT) probes are designed to accommodate a wide range of inspection scenarios and conditions, each tailored to address specific challenges and environments. From inspecting planar metallic surfaces to complex geometry, internal surfaces of tubes, and high-speed or high-temperature applications, ECT probes are engineered for precision and adaptability. The orientation of the eddy currents (EC), particularly their transversal alignment to defects, plays a crucial role in maximizing detection sensitivity, as depicted in Figure 7. Consequently, the shape and design of the coils are paramount in determining the efficacy of the inspection. This section delves into the diverse array of ECT probes and systems developed for various inspection needs, emphasizing the importance of the coil orientation and its impact on defect detection.

### 3.1. ECT Probe Solutions for Planar or Linear Inspections

**Figure 8:**
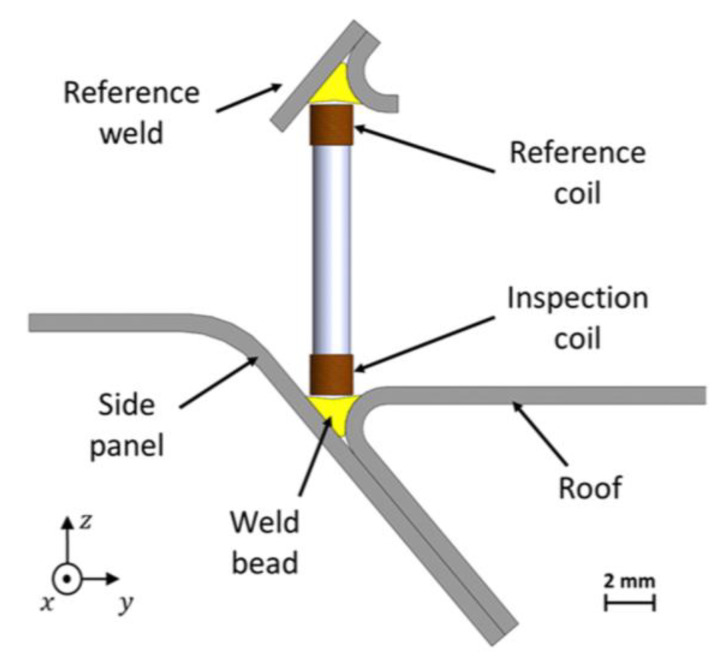
Two cylindrical helicoidal bobbin coils operating in bridge differential mode. One of the bobbins moves over the weld bead, while the other has the opposite orientation, with a reference weld in good conditions [55].

Traditional eddy current testing uses a pancake circular-shaped excitation coil, as shown in Figure 5a. It causes a circular eddy current flow pattern. This design provides strong induction but is susceptible to lift-off effects from uneven surfaces, leading to impedance changes and potential false readings. Additionally, defects parallel to the eddy current distribution are often overlooked [136,137,138]. In addressing the challenges of the lift-off variation, particularly in measuring the metallic plate thickness, Yin et al. designed a triple-coil sensor operating in a multifrequency mode. The sensor consisted of three helicoidal circular coils, all of the same dimensions, arranged coaxially and spaced equally. This design allowed the sensor to function as two distinct coil pairs: the bottom and middle coils as one pair, and the middle and top coils as another. By positioning the second pair further from the test sample, it effectively simulated a higher lift-off while maintaining a constant lift-off difference between the two pairs throughout the operation. Measurements were conducted by exciting the middle coil and capturing induced voltages from the bottom and top coils. This approach not only saved time by allowing simultaneous measurements, but also reduced measurement errors associated with switching between coil pairs. The method demonstrated strong immunity to lift-off variations, a critical advantage given that such variations are often unavoidable in practical settings [73]. Machado et al. developed an ECT system for inspecting automobile laser-brazed welds, featuring customized probes, digital ECT instrumentation, and a robotized arm for automated inspections. They used cylindrical helical probes with ferrite cores and small dimensions. The bobbin coils had an outer diameter of 2 mm and a height of 1.65 mm, while the weld bead profile was around 2.3 mm in width. The probes consisted of two cylindrical helicoidal bobbin coils operating in a bridge differential mode, enhancing sensitivity by comparing the inspected weld with a reference weld in good condition (Figure 8). Additionally, the operation at two frequencies, high and low, allowed for the detection and differentiation of surface and sub-surface defects [55,56].

Janousek et al. proposed a novel method to enhance crack sizing capabilities in eddy current non-destructive testing. This approach involved using multiple probes driven at the same frequency, but generating different eddy current distributions. They utilized two mutual-inductance-type eddy current probes, each consisting of two exciting coils and one pick-up coil. The exciting coils, coaxially rectangular and tangentially positioned relative to the test surface, drove the eddy currents. A pancake pick-up coil located between the exciting coils sensed the signals, which were then linearly superposed. The feature value of the superposition ratio provided a clear indication of the crack’s depth. This method demonstrated the ability to size cracks deeper than the standard penetration depth, using notches measuring 40 mm in length, 0.5 mm in width, and 10 to 20 mm in depth, introduced into a SUS316L plate specimen with a thickness of 25 mm. This approach represented a significant step forward in the precision and effectiveness of ECT for detecting and sizing cracks [139]. Pereira et al. developed and validated a fast and accurate method of computer-aided sensor design, focusing on six defect types commonly found in weld overlay Inconel claddings. They considered a superficial sensor with two coaxial coils operating in the transmit-receive mode, simulated using COMSOL Multiphysics. The prototype was tested on identical blocks, showing excellent agreement, validating this robust design strategy [140]. Tytko et al. advanced flaw detection in conductive materials by replacing the traditional air-cored coil model with an ideal filamentary coil. This adaptation not only simplified the calculations, but also enhanced the design and calibration processes for both magnetic and non-magnetic materials [141,142]. They developed an analytical model of a filamentary coil positioned above a three-layer plate with a hole, which achieved accuracy comparable to that of air-cored coils. This approach facilitated the modeling of various defect types and was validated through experiments and finite element method (FEM) simulations [142].

**Figure 9:**
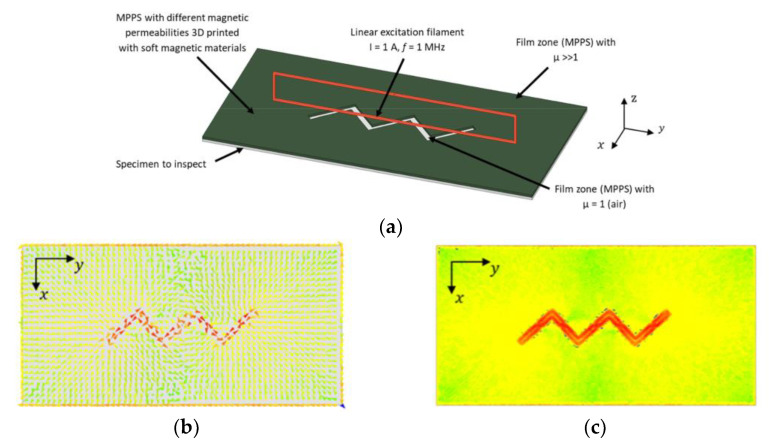
Zigzag-shaped MPPS cutout pattern on an aluminum specimen: (**a**) simulation model; (**b**) EC vector field simulation results; (**c**) EC density simulation results [135].

In contrast to the widely utilized circular probe coils, rectangular coils have emerged in eddy current inspections of plates, offering notable advantages in certain applications [143,144]. These rectangular coils are particularly effective in crack detection due to their directional properties and their ability to create more uniform eddy current distributions compared to circular coils [145]. Rosado et al. introduced a new eddy current probe design aimed at detecting small surface-breaking defects in friction stir welding (FSW) aluminum joints [146]. This probe features a planar disposition and operates on a differential basis, which significantly enhances its sensitivity to defects aligned with specific orientations, such as those found along welded joints [75]. The probe generates eddy currents by flowing an alternating current through a driver trace located symmetrically between two sensitive D-shaped spiral coils. The sensing of the magnetic field is achieved through these two coils, which share a terminal. The probe, produced using printed circuit board (PCB) technology on a single substrate, measures the sum of induced voltages from the coils. In symmetrical conditions, these voltages are equal in magnitude but out of phase, resulting in a net output of zero. The presence of defects alters the magnetic field and the voltage equilibrium, leading to a non-zero output voltage. Defect detection is based on characterizing the complex ratio between the output voltage on the sensing coils and the input current in the driver trace [147]. Finite element modeling was employed to study the probe’s operation and assess the defect’s impact on its response. Experimental validation confirmed a good agreement between simulated and measured responses for various defects, demonstrating the accuracy of the simulation model [148]. The probe successfully detected root defects as small as 60 μm [149]. However, the original design faced limitations in detecting defects oriented perpendicular to the sensitivity axis. To address this, an improved probe structure was developed, featuring additional driver and sensing elements. This new design includes four driver traces forming a cross pattern with four sensing coils, enabling the modification of the eddy current pattern during testing. The sensing coils are arranged with alternate winding directions to enhance the defect detection capabilities [150]. ECT often struggled to detect defects aligned parallel to the primary field direction of the static magnetic fields. To overcome this limitation, researchers explored the use of rotating field techniques in ECT probe design. One notable advancement in this area was the development of the rotating focused-field eddy current sensing technique. This method involved rotating the magnetic field generated by the probe to improve defect detection. The rotation was achieved using multiple coils arranged in specific configurations within the probe, with the controlled energization of these coils producing a rotating magnetic field around the probe [151]. This rotating field enabled the detection of defects in various orientations, enhancing both the sensitivity and accuracy. This technique proved especially effective for inspecting complex geometries and materials with anisotropic properties, offering improved resolution and clarity by concentrating the field in specific areas. Consequently, it provided a more comprehensive inspection solution, particularly in cases where traditional static field ECT might miss certain defect orientations [152,153]. Building on this concept, Xu et al. further advanced rotating field techniques by developing a sensor with four identical excitation coils arranged in an inverted pyramid configuration, combined with a giant magneto-resistive (GMR) detection element. These coils formed two Figure 8-shaped focusing sub-probes, driven by two identical harmonic currents with a 90-degree phase difference. This innovative design exemplified the effective application of rotating focused field techniques, significantly enhancing the ability to detect defects oriented arbitrarily and broadening the range of ECT applications [154]. Ge et al. proposed an innovative approach to translating the outcomes of rotating eddy current testing (RECT) into results that reflect uniform eddy current testing for specific orientations. Their ECT probe featured two rectangular orthogonal exciting coils paired with a circular pickup coil. This design aimed to enhance detection capabilities and broaden the applicability of RECT in directional non-destructive testing scenarios. However, this study found that additional currents induced by RECT could introduce noise. Therefore, careful signal processing and analysis became essential to account for the orientation of the induced eddy currents [155]. Chen et al. proposed a planar eddy current NDT probe based on Koch curve fractal geometry excitation coils to improve the probe sensitivity by inducing multi-radius eddy currents in the conductive material. Traditional circular excitation coil probes struggle to detect cracks significantly smaller than the radius of the excitation coils [156]. Thus, the Koch curve was used to design the new excitation coil geometry that can induce a multi-radius EC in the test sample [157]. To address this, Chen et al. employed Koch curve fractal geometry to design a new excitation coil that induces smaller-radius eddy currents. The probe, which integrates Koch curve fractal geometry excitation coils and circular pick-up coils on a Four-layer PCB, demonstrated improved detection capabilities for defects shorter than the sensor’s size without reducing the sensor size itself [158,159,160]. However, the probe’s absolute sensor design made it susceptible to lift-off noise, and its spatial resolution was limited due to the 18 mm sensor size. To overcome these limitations, a differential Koch coil exciting planar EC probe was proposed [161], and further developments in higher-dimensional Koch curve fractal geometries have shown promise in enhancing detection sensitivity for defects at various depths and angles [162]. She et al. introduced a multiple floral eddy current probe made from flexible PCB material to address the sensitivity to lift-off distance variations while maintaining high accuracy and sensitivity. This probe features a planar arrangement of helicoidal circular coils, with a central reception coil surrounded by several transmission coils. To minimize the interference from the magnetic field generated by the transmission coils, the TX coils are connected sequentially in reverse order, and the excitation currents in adjacent TX coils flow in opposite directions, thereby cancelling out the induced magnetic fields in the RX coil. This design resulted in improved sensitivity and a significantly reduced the lift-off effect. The probe was validated on an aluminum plate and its flexibility suggests potential benefits for non-planar surfaces, though further testing on such surfaces is needed [163]. Machado et al. explored the innovative use of high magnetic permeability substrates, created through additive manufacturing, to shape eddy currents for NDT applications. Traditional EC probes often rely on complex coil geometries to direct currents perpendicular to defects, enhancing detection but posing manufacturing challenges. Machado’s approach shifts this complexity to the magnetic substrate, simplifying the coil design while leveraging the advantages of additive manufacturing. Figure 9 shows how a simple linear coil can generate a zigzag pattern EC flow using this technology. Although the study showed promising results, practical implementation in real-world industrial settings requires further validation. Future research should focus on long-term durability, the impact of different materials, and the scalability of the additive manufacturing process for mass production [135].

Brun et al. proposed an innovative solution for integrating sensors directly onto the parts being inspected, addressing issues of reproducibility and minimizing human error. This method also proves advantageous for use in confined or hazardous environments. The study explored two printing techniques on flexible substrates: dispenser printing and screen printing. Dispenser printing involved using an ink syringe to deposit conductive ink onto a substrate. This method successfully created a 10-turn spiral coil within a 150 mm^2^ area. In contrast, screen printing employs a mesh to transfer ink onto the substrate, with a blocking stencil preventing ink in specific areas. This technique offers benefits such as enhanced reproducibility and higher resolution, which enables the creation of more turns within a given area. The effectiveness of these printed sensors was validated through the detection of a small diameter hole using a permanently printed sensor. These sensors demonstrated good adhesion to the test parts and provided electromagnetic responses comparable to traditional portable sensors. However, for structural monitoring applications, further development is needed to ensure the long-term viability of these devices. Additionally, the performance of the printed sensors compared to flexible PCBs remains unclear, suggesting that more research is necessary to fully assess their effectiveness and durability in practical scenarios [164].

### 3.2. ECT Probe Solutions for Complex/Curved Parts

**Figure 10:**
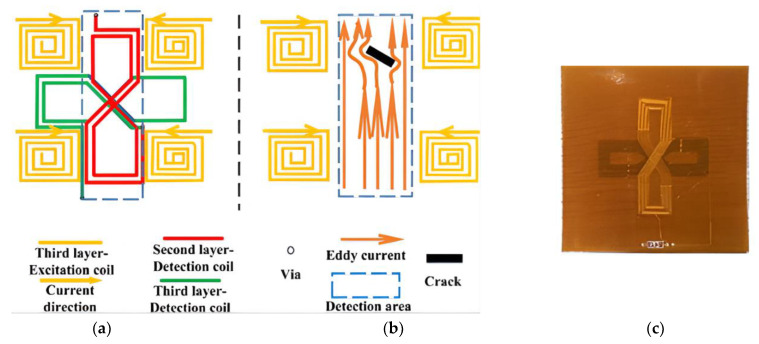
Schematic diagram of the proposed probe structure (**a**) and EC interaction with crack (**b**); (**c**) PCB of the pick-up coil [166].

Inspecting cracks on complex curved surfaces presents significant challenges in ECT, particularly concerning lift-off immunity and the probe’s detection capabilities [165]. Li et al. tackled these challenges by designing a flexible differential eddy current probe specifically for inspecting cracks on complex curved surfaces. Their probe, fabricated using flexible printed circuit technology, features a multilayered structure that adapts to the geometry of irregular surfaces. It includes a driver coil located on the third layer and a pick-up coil situated across the first and second layers. The driver coil consists of square coils arranged at the probe’s corners, while the pick-up coil comprises two 8-shaped coils oriented orthogonally, as shown in Figure 10. This configuration, based on the transmission model, improves the detection of cracks with varying orientations and addresses lift-off challenges by conforming to the surface geometry of objects like rail treads. Both simulation and experimental validations demonstrated that this probe is effective at detecting cracks of different orientations on complex curved surfaces [166].

Zhang et al. developed a flexible eddy current array probe designed for scanning defects on the last-stage blades of steam turbines, utilizing a Cartesian coordinate robot. Steam turbine low-pressure rotor blades are subjected to extreme conditions, including high rotational forces, elevated temperatures, corrosion, and erosion during operation. The proposed flexible EC array sensor addresses these challenges by offering flexibility and adaptability. Unlike traditional eddy current sensors, this array probe can be bent or folded, accommodating the complex geometries of turbine blades. Standard inductors, rather than printed traces, were used in the coil design to achieve a higher number of windings, though this resulted in an increased probe height. The probe’s effectiveness is demonstrated through signal processing and fitting equations that account for height and inductance changes, which allows for accurate three-dimensional imaging of the blade surface. The experimental results validated the probe’s ability to detect and image defects such as erosion, pitting, and edge defects on the blade surface [167]. Similarly, Xie et al. introduced a novel flexible eddy current array specifically designed for measuring fatigue crack lengths. This array features a transmission/reception (T/R) coil structure with etched coils on polyimide film, employing flexible printed circuit board (PCB) technology. The array includes a large uniform exciting coil and 64 sensing coil elements arranged in an inclined zig-zag orientation, providing a spatial resolution of 0.8 mm and an effective coverage length exceeding 50 mm. The four-layer structure of the sensor array includes exciting coils on the top and bottom layers, with sensing coils on the middle layers, interconnected via bridges. This flexible design facilitates close contact between the sensor and complex surfaces, improving detection capabilities. Finite element simulations using AC/DC module in the COMSOL Multiphysics software were conducted to evaluate the sensor’s performance, and the experimental results confirmed that the array is sensitive to microcracks and capable of accurately sizing crack lengths. The consistent alignment of the experimental and simulated results underscores the array’s effectiveness in detecting and measuring fatigue cracks [168]. In another innovative approach, She et al. developed a flexible differential butterfly-shaped array of eddy current sensors designed specifically for detecting defects on the surface of iron screw threads. The sensor comprises a butterfly-shaped coil, which consists of two rectangular coils connected at the center by a bridge, and additional sets of differential planar circular helical RX coils, along with a top RX coil positioned over the bridge. This configuration enhances the sensitivity by addressing the lift-off problem commonly encountered in screw thread defect detection. Both the simulation and experimental validations confirm the sensor’s high performance and low error rate, demonstrating its capability to detect defects as small as 0.35 mm and 0.22 mm on iron screw thread surfaces effectively [169]. Zhang et al. introduced a novel in-plane differential coil array probe on a flexible PCB that operates at high frequencies with exceptional sensitivity. The probe features 16 coils arranged perpendicularly to the PCB plane, with a four-layer structure and a total of 16 turns per coil. It covers a rectangular area of 52.5 mm in width and uses a differential setup to minimize background signal interference, with only one pair of coils active at a time. Testing on turbine blades and CFRP tubes showed the probe’s capability to detect defects with various orientations and micro-dimensions effectively. However, further improvements are needed, including the optimization of coil parameters and advanced image processing algorithms for field applications [170].

### 3.3. ECT Probe Solutions for Holes Inspection

The timely detection of fatigue cracks is crucial for ensuring the fail-safe operation of long-term exploited aircraft structures and for implementing damage tolerance approaches. ECT has been introduced in the aircraft industry as a method for inspecting cracks in bolt and rivet holes [171,172]. To enhance the detection and characterization of cracks around fastener holes in multilayer structures without the need to remove the fasteners, Knopp proposed model-based approaches that support the design of advanced EC systems. His work validated and applied models to simulate EC inspections as part of the design process [173]. Joubert et al. developed an EC probe designed to rapidly and accurately capture C-scan images related to surface-breaking defects. Given the cylindrical geometry of the components being inspected, the probe featured a global inducer composed of a large coil coaxial with the bore hole, facilitating effective electromagnetic coupling with the part. This configuration induced a uniformly oriented eddy current flow within the inner wall of the bore hole. In the absence of defects, symmetry ensured that no radial component of the magnetic field was generated. However, the presence of a surface-breaking defect altered the eddy current flow locally. The inducer was designed as a single-layer bobbin coil, measuring 58 mm in length and 48.8 mm in diameter, featuring 76 turns wound with 1.25 mm diameter copper wire to minimize the capacitance. The sensing array comprised pickup coils that were 1.4 mm long and had an outer diameter of 1.4 mm, featuring 460 turns distributed across 10 layers of 46 turns, wound with 50 μm-diameter copper wire. The test subject was a bore hole with a diameter of 52 mm, machined into a 170 mm × 100 mm × 50 mm mock-up made from 2024 T3 aluminum alloy. The mock-up contained three defects, labeled D1, D2, and D3, which were machined using an electrical discharge machining (EDM) process. These defects were semi-circular, with a 200 μm aperture, and diameters of 0.4 mm, 0.8 mm, and 2 mm, respectively. The experimental results demonstrated the probe’s effective sensing capability within the 10–800 kHz frequency range, achieving a peak signal-to-noise ratio (PSNR) higher than 36 dB for defects as small as 0.4 mm in diameter [174]. To further enhance the sensitivity, Chen proposed a rosette-like eddy current array sensor. This design utilized a driver pickup coil probe system with an array configuration that allowed for same-direction exciting currents, thereby preventing local eddy current loops that could disturb the measurements. The layout of the pickup coils improved the angular resolution of the sensor, making the eddy current distribution more sensitive to defect propagation [175]. Shao et al. developed an automatic system for detecting rivet hole defects in aircraft structures using a custom EC sensor array and image analysis algorithm. The multi-channel sensor array, with sixteen coils wound with copper wire arranged in two staggered rows, ensures the continuous coverage of the sensing area. Each coil contains 200 turns of wire. The system, tested on a 2 mm-thick aluminum plate with 5 mm-diameter holes, including one with a crack, used simulations with ANSYS MAXWELL software to optimize the sensitivity and penetration depth. The system successfully detected and located fatigue cracks, providing an automatic, real-time, and accurate inspection method for the riveted joints [58].

### 3.4. ECT Probe Solutions for Tube Inspection

**Figure 11:**
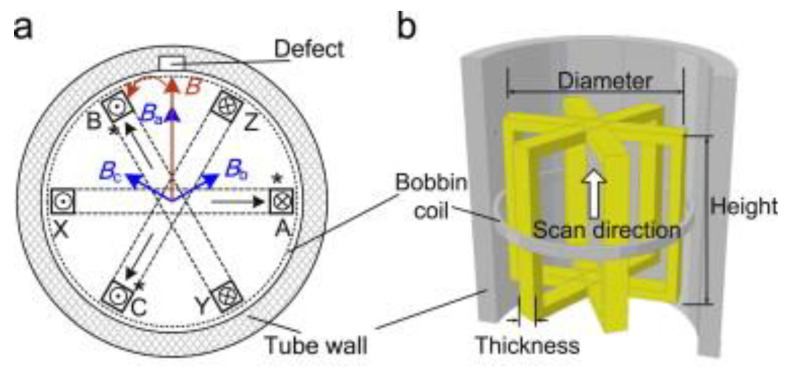
Schematic Rotating field windings and bobbin pickup coil: (**a**) three-phase windings arrangement and (**b**) 3D model of bobbin pickup and three windings inside the tube [185].

Conventional bobbin probes used for inner pipe inspections typically feature circumferential windings that induce circumferential eddy currents. In this configuration, defects oriented transversely to the direction of the eddy currents generate output signals with a greater amplitude, whereas defects aligned parallel to the eddy current direction yield smaller output signals [176]. In exploring analytical solutions for pipe inspections, several methods have been proposed to optimize the detection capabilities. These solutions often involve mathematical modeling to predict eddy current behavior in various scenarios, contributing to the design of more effective inspection systems [177,178]. Huang et al. developed an arrayed multi-coil probe specifically for testing and sizing cracks in steam generator tubes. This innovative probe utilizes a multi-coil arrangement that facilitates rapid detection across the entire tube without the necessity for rotation. The design consists of a simple configuration of 3 × 10 circular coils, each with a diameter of 2 mm. Various exciting and sensing patterns can be employed, allowing the coils to function as either drivers or pickup coils. Four distinct patterns were examined, although the experimental results indicated that while the probe demonstrated high detectability, the optimal pattern for performance remained unclear [179]. Sun et al. proposed a flexible arrayed eddy current sensor to inspect the hollow axle inner surface of a high-speed train [180]. Four-layered flexible PCB with excitation traces and sensing traces was rolled and mounted on the sensor holder which was 28.6 mm in diameter. The sensor was configured as a transmit/receive type. The sensor consisted of 28 rectangular sensing traces and two independent excitation traces with the same alternating current flowing that traveled around the outer perimeter of the sensing coils. The results from the simulations and experiments show that the sensor is capable of detecting both longitudinal and transverse defects with depths as small as 0.5 mm. However, the sensor is more sensitive to the transverse defects [181]. In another advancement, Machado et al. experimentally validated a probe for inspecting the inner surfaces of austenitic steel jackets used in the ITER project, focusing on improving the detectability of circumferential defects. Their proposed linear array of trapezoidal spiral sensing coils effectively eliminated the blind zones associated with traditional circular spiral coil arrays. This design enhanced the accuracy of the defect location in the circumferential direction, as a single array with N coils can distinguish between 2N regions. The use of a flexible substrate allowed the coils to be positioned closer to the tube surface, thereby increasing the sensitivity. The excitation coil was twisted at an angle to enhance the disturbance of the eddy currents caused by both circumferential and axial defects. The pickup mechanism incorporated an array of planar trapezoidal spiral coils arranged on a flexible substrate around a cylindrical chassis, with the sensitive plane oriented perpendicular to the radial direction. The number of coils and their dimensions can be adjusted based on the desired spatial resolution; increasing the number of coils leads to an improved resolution. The probes demonstrated superior sensitivity, successfully detecting defects with a depth of 0.5 mm and a thickness of 0.2 mm [63,182]. Bobbin coils and bobbin-type Hall sensor arrays have been proposed as alternatives for crack inspections within small-bore piping systems. This method enables the high-speed imaging of cracks without the need for a scanner, as the electromagnetic (EM) field is distorted by the presence of defects. An array comprising 32 × 32 Hall sensors, achieving a spatial resolution of 0.78 mm, was embedded in a cylinder with a diameter of 15 mm and a length of 24.96 mm. A bobbin coil operating at 5 kHz of alternating current was placed inside the piping system, while the sensor array was positioned externally. This configuration was evaluated using specimens made of titanium alloy, with simulations conducted using finite element modeling (FEM) in ANSYS [183]. To overcome the limitations associated with conventional probes, various innovative approaches have been explored. One such method involved the inclination of the bobbin windings, allowing for different orientations between the pickup and excitation coils—these orientations can be parallel, symmetric, or twisted. This design alteration meant that the eddy currents were no longer aligned strictly with the circumferential direction, leading to improved sensitivity in detecting defects. However, challenges remained; in certain circumferential positions, defects could still align parallel to the coils, potentially leading to missed detections without the mechanical rotation of the probe [184]. Another noteworthy approach for pipe inspection is the use of a rotating-field eddy current probe featuring a bobbin pickup coil that generates a rotating magnetic field, thereby negating the need for mechanical rotation [185,186,187,188]. This design utilized three identical coils positioned at 120° angles, powered by a balanced three-phase source [189]. The vector sum of the magnetic fields produced a circumferentially rotating field around the pipe. The validation of the probe involved artificial defects characterized by through-wall square holes measuring 3.5 × 4 mm^2^ and 4 × 4 mm^2^ in Inconel^®^ 600 pipes, which have a conductivity of 9.69 × 10^5^ S/m. The probe demonstrated sensitivity to defects of all orientations, allowing for the estimation of both the depth and location from a single line scan. Subsequently, the research group enhanced the probe design by integrating a giant magneto-resistive (GMR) sensor, yielding promising results. This prototype was sensitive to both axial and circumferential notches, with C-scan imaging clearly delineating the defect location and orientation [190]. Figure 11a shows the magnetic flux density components Ba, Bb, and Bc, associated with three windings (AX, BY, and CZ), oriented perpendicular to the plane of each winding.

These components combine to create a total magnetic flux density vector B, which maintains a constant amplitude over time while rotating in sync with the excitation source. In the cross-section of the pipe, the magnetic field primarily rotates radially, circulating around the radial axis, thus enhancing sensitivity to cracks of all orientations. When no defect is present, the magnetic flux predominantly flows in the radial direction, with no axial magnetic flux evident. However, the introduction of a defect near the center plane disrupts the radial magnetic fields, resulting in the emergence of an axial magnetic field component [191]. Daura et al. developed a transmitter–receiver (Tx–Rx) flexible printed coil (FPC) array utilizing a wireless power transfer (WPT) approach with dual resonance responses. This innovative design allows for the extraction of multiple parameters from samples, defect characteristics, lift-offs, and material properties. The flexibility of the coil array facilitates the area mapping of complex structures. To validate this method, experimental investigations were conducted using a single excitation coil coupled with multiple receiving coils based on the WPT principle. These tests were performed on the curved surface of a pipe exhibiting a natural dent defect. The FPC array comprised one excitation coil and 16 receiving coils, which were employed to measure the dent by collecting data from 21 C-scan points on the designated sample. The gathered experimental data served as a foundation for training and evaluating the dual resonance responses concerning multiple feature extractions, selections, and fusions aimed at a quantitative non-destructive evaluation (NDE). Four specific features were investigated, including the resonant magnitudes and principal components of the two resonant areas. These features were analyzed through correlation analysis to facilitate feature selection and fusion using deep learning techniques. The results indicated that deep learning-based multiple-feature fusion significantly enhanced the performance of 3D defect reconstruction in WPT-based flexible printed coil eddy current testing (FPC-ECT). This approach showcased the potential for advanced defect characterization and mapping in complex geometries, emphasizing the benefits of integrating modern machine learning techniques in NDE applications [192].

### 3.5. ECT Probe Solutions for Wire Inspection

The steel wire rope serves as a critical tensile and load-bearing component, extensively utilized across major industries, including agriculture and services [193]. However, when employing ECT for detecting broken wires in spiral ropes, the alternating peaks and valleys of the rope surface can complicate defect identification. Cao et al. designed an adjustable, annular testing device featuring probes arranged in radial symmetry, leveraging low-frequency transmission eddy current testing. This innovative device aims to address common limitations associated with eddy current techniques, such as the lift-off effect, edge effect, and skin effect. Specifically, the lift-off effect is mitigated by adjusting the spacing between the two probes, while the appropriate selection of the excitation frequency effectively avoids the skin effect. Additionally, the mechanical adjustment of the circumferential position of the probes within a combined bracket transforms the edge effect into a lift-off effect, minimizing its adverse impact on damage detection, provided that the edge remains constant [194]. Steel wire ropes, constructed from high-carbon steel wire, typically consist of several strands wound around a central core. The variability in lift-off between the bottom of the coil and the rope surface during scanning detection results in a sinusoidal variation of the output voltage [195]. Therefore, the influence of the spiral structure on the eddy current signal induced by broken wires is a critical consideration. To enhance the detection of broken wires, Yanfei developed an eddy current array differential probe. This probe features a pair of symmetrically placed coils that are differentially connected, with the detection signal being the output voltage of the differential bridge [196]. When scanning over an intact section of the rope, the surface beneath the coils exhibits symmetry, resulting in a zero-output voltage. However, the presence of broken wires disturbs this symmetry, leading to a non-zero output voltage above the damaged areas. By analyzing the voltage output from the differential bridge, it becomes possible to detect surface-level broken wires. The design effectively mitigates external disturbances, such as a temperature drift and the inherent characteristics of the wire rope’s peaks and valleys. As the probe passes over broken wires, a significant increase in the output voltage is observed. The scanning signal is then processed using a wavelet-based denoising method to enhance the signal-to-noise ratio. The experimental results demonstrate that the proposed method effectively identifies the extent of damage in surface-broken steel wires. The simulation results further confirm that the influence of the alternating peaks and valleys of the rope surface on the eddy current response signal of broken wires can be effectively eliminated through the use of the proposed eddy current differential probe [196].

### 3.6. ECT Probe Solutions for High-Temperature Applications

**Figure 12:**
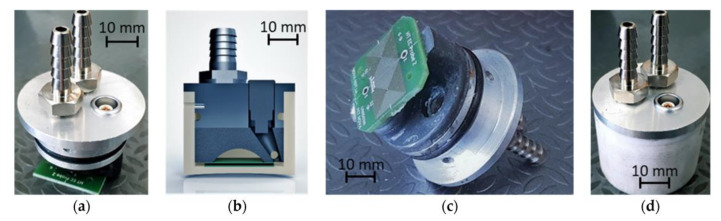
Construction and chassis components for a high-temperature pipe base material inspection probe. (**a**) Assembled PCB probe with lid; (**b**) CAD cross-section of the probe; (**c**) bottom view of the assembled PCB probe and lid; and (**d**) complete probe assembly [51].

One notable application in the realm of in-service high-temperature component inspection is the monitoring of steam transportation pipelines, which must endure temperatures exceeding 300 °C [197]. This necessitates the development of tailored inspection and condition monitoring methods suitable for such extreme conditions. While several solutions exist for pipe inspections, examples specifically addressing high-temperature applications and customized equipment remain scarce [198,199,200,201]. The literature highlights various high-temperature NDT applications, including the permanent inspection of hot wire [198] and in situ monitoring using techniques like eddy currents [202] and ultrasound [203,204]. Unlike acoustic properties, the electromagnetic characteristics of materials exhibit less variability with temperature, presenting a unique challenge for ECT. The primary concerns in high-temperature ECT involve ensuring the proper thermal isolation of the probe and managing the resulting lift-off effect. Research has documented the temperature’s influence on ECT measurements, with some studies focusing on characterizing and compensating for minor temperature variations to enhance the measurement accuracy [205]. For instance, the inspection of fuel rods and plates in nuclear reactors has been studied, where temperature effects were modeled using empirical functions [206]. In other cases, parts experience significant temperature fluctuations during processes such as heat treatment [207]. The capacity to conduct high-temperature inspections facilitates the continuous monitoring of industrial components during regular operations, thereby minimizing the downtime and associated costs. Santos et al. developed an automated NDT system specifically for the in-service inspection of orbital welds on tubular components operating at temperatures as high as 200 °C [51]. This system integrates ultrasonic and eddy current techniques, incorporating specialized strategies to address high-temperature conditions. In their design, eddy currents were utilized to detect surface and subsurface cracks. The testing involved a standard block with a tubular geometry featuring two butt welds, constructed from P235GH steel, which is well-suited to high-temperature and high-pressure applications. Two customized ECT probe prototypes were created: one aimed at detecting defects in the pipe’s base material and the other focused on weld bead defects. The first probe, depicted in Figure 12, featured two rectangular planar coils operating in a differential bridge mode, which enhanced the sensitivity through closer contact with the inspected material. These coils were fabricated using printed circuit board (PCB) technology, with a high-performance FR-4 epoxy core selected for its glass transition temperature of 180 °C, making it suitable for prolonged high-temperature inspections [51].

The second probe, designed for weld bead defect detection, also employed two coils in the bridge-differential mode, ensuring consistent positioning along the weld bead. This configuration minimized the probe’s response to variations in the weld bead profile, which could otherwise obscure the detection of smaller defects. The innovative design included orthogonal crossing coils, commonly used in inspecting other welded components. To facilitate the high-temperature operation, a water-cooled chassis was developed, comprising an insulating cup and a metallic cover. The probe was secured to an aluminum cover featuring connectors and water inlets for cooling. The insulating cup was crafted from machined Teflon, enhancing the thermal management. Despite the elevated lift-off required for high-temperature ECT, both differential probes demonstrated an impressive performance, with the PCB probe achieving a higher signal-to-noise ratio. Importantly, high temperatures did not adversely affect the results, attributed to the effective cooling systems in place, with an experimental validation showing a negligible impact even at temperatures reaching 300 °C [51].

### 3.7. ECT Probe Solutions for Additive Manufacturing

Additive manufacturing (AM) is a cutting-edge production technique that builds parts by sequentially fusing thin material layers to create three-dimensional products of desired sizes and shapes [208,209]. Initially used mainly for rapid prototyping to speed up model development, AM has since evolved to produce functional parts, including those made from metals. This development opens up opportunities for using ECT to inspect and monitor these parts. ECT, leveraging electromagnetism, is particularly well-suited to evaluating surface and subsurface layers in metal AM products, making it ideal for detecting defects, especially when performed inline after each deposited layer [210].

Selective Laser Melting (SLM) is a specific AM technology that uses a high-power laser to transform metal powder into solid layers [211,212,213,214]. Geľatko et al. investigated the effectiveness of ECT for identifying defects in AM stainless steel parts and analyzed variations in eddy current data due to various artificial defects, using absolute cylindrical helicoidal probes [210]. Du, W, et al. [48], tested an ECT device for detecting subsurface defects in a Ti-6Al-4V part produced by additive/subtractive hybrid manufacturing (ASHM), which aims to enhance the quality of printed parts. This study, along with similar research on SLM Inconel 738LC alloy using a differential EC probe with two oppositely wound coils, demonstrated high sensitivity to defect-induced magnetic field changes. The sample was heated from 25 to 300 °C to simulate real-world conditions, revealing that edge effects significantly impacted the ECT results [215]. Duarte et al. compared various non-destructive testing techniques, including ECT, for defect identification in SLM-manufactured parts [23]. Farag et al. investigated two distinct eddy current probe designs to assess their effectiveness in detecting artificial defects in parts made from stainless steel (316) and titanium (Ti-6Al-4V). The study involved two types of probes. One type was a circular helicoidal coil probe operating in absolute mode. This design proved particularly effective in detecting notches, making it well-suited for identifying defects such as cracks and incomplete fusion holes in the material. The second type was a reflection probe, which featured two concentric cylindrical helicoidal coils. In this configuration, the outer coil functioned as the pickup coil, while the inner coil served as the driver coil. This reflection probe was found to be more effective in detecting small-diameter blind holes, particularly those with diameters of less than 0.2 mm. It demonstrated a superior performance in identifying these tiny defects compared to the absolute probe [216]. In a separate study, Spurek et al. performed in situ monitoring of metals during powder bed fusion (PBF) using ECT. The ECT equipment was integrated into a commercial PBF machine to enable the layer-by-layer monitoring of the relative density of parts as they were being produced. The ECT probe used was a commercial ferrite pot core coil configured in a bridge setup, which included an identical coil for balancing. The system operated in absolute mode, and the measurement data revealed that layer-to-layer differences at a relative density of about 0.1% could be effectively detected using ECT. This demonstrated the probe’s capability to monitor and assess the quality of parts in real-time during the additive manufacturing process [217]. In a related development, Barrancos et al. introduced a novel eddy current testing array probe and associated readout electronics aimed at improving layer-wise quality control in metal additive manufacturing using powder bed fusion. Their proposed design approach focused on several key benefits, including the scalability of sensor numbers, exploration of alternative sensor elements, and minimalistic signal generation and demodulation techniques [218]. The solutions proposed were intended to enhance one-dimensional ECT array probes by adjusting sensor pitch and readout speed to enable effective layer-wise imaging while being installed on the recoater units of PBF machinery [218]. One significant aspect of their work was the use of mass-produced, commercially available discrete surface-mounted device (SMD) coils as an alternative to custom-made coils and magneto-resistive (MR) sensors. The motivation for this choice was based on the low cost, design flexibility, and ease of integration with readout electronics [219]. To improve the measurement sensitivity, the researchers preferred absolute ECT coil measurements, which directly relate to surface conductivity. To address the challenge of low impedance sensitivity, they employed a compensation coil wired in a bridge-differential configuration. The coils were tested on a reference feature with a 0.8 mm diameter and 0.8 mm depth hole in a stainless steel 316 part produced via laser powder bed fusion (LPBF). Various coil models were evaluated to optimize their sensitivity while maintaining high spatial resolution. The findings confirmed that wire-wound, ferrite-cored inductors are a viable option for ECT sensor implementation. Although the study did not produce a large ECT array probe, the specifications of the developed probe and its electronics achieved the necessary acquisition speed for effective online, layer-wise imaging during the PBF process [218]. Saddoud et al. developed two new ECT probes specifically for inspecting stainless steel 316 L mock-ups produced with powder bed fusion. One probe was designed to detect notches as small as 1 mm in length, 0.3 mm in width, and 0.1 mm in depth, while the other was intended for detecting engraved letters with a depth of 1 mm. Using the CIVA non-destructive testing software package for simulations, several sensor designs and their parameters were tested to determine the most optimal configurations. Ultimately, they designed two types of sensors: a separate transmitter/receiver sensor and an isotropic sensor [220]. The separate transmitter/receiver sensor features an overlapping design consisting of a transmitter coil and a receiver coil etched onto a Kapton flexible film (thickness 0.07 mm). In this design, one coil is used for excitation while the other receives the signal from the part under test. This arrangement enhances the defect signal in the impedance plane for homogeneous planar parts [221]. Although Kapton film allows for the inspection of parts with complex geometries, it was not utilized for typical L-PBF (laser powder bed fusion) technique-fabricated parts. The overlapping design helps mitigate variations in lift-off between the two coils, improving defect signal detection. The isotropic sensor pattern consists of a transmitting coil and two receiving coils, also engraved on Kapton film. The transmitting coil is placed on top, while the two receiving coils are disposed coaxially, one on the inside and the other on the outside. The winding direction of the coils alters their polarity. The reference winding direction is aligned with the transmitting coil, and the receiving coils can be designed either in the same or opposite winding direction as the transmitting coil. The comparison results indicated that the isotropic sensor provides a better spatial resolution and signal-to-noise ratio (SNR) for detecting letters. For defect detection, particularly when defects are aligned with the scanning direction (which is optimal for eddy current detection), the separate transmitter/receiver sensor offers a superior SNR. However, for detecting defects with unknown orientations, the isotropic sensor proves to be more effective [220].

**Figure 13:**
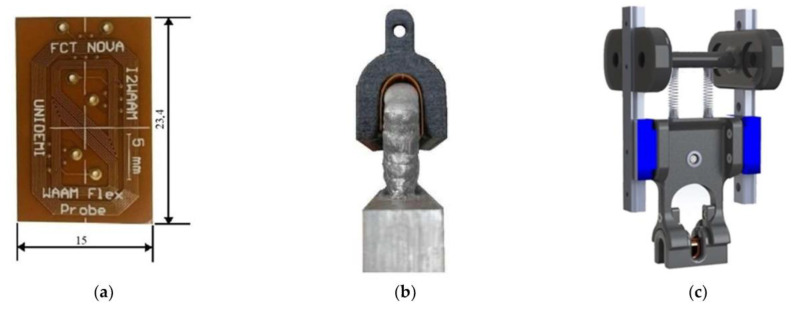
Multi-layer flexible PCB probe for WAAM walls: (**a**) flexible PCB; (**b**) PCB assembled in a flexible TPU chassis, mounted on a WAAM wall specimen; and (**c**) complete probe setup system [226].

Wire arc additive manufacturing (WAAM) falls under the category of directed energy deposition (DED-arc) and involves using an electric arc as a heat source combined with a wire as the feedstock material. The process builds upon established welding techniques such as gas metal arc welding (GMAW) [222], plasma arc welding (PAW) [223], and gas tungsten arc welding (GTAW) [224]. Bento et al. developed an ECT probe for the layer-by-layer monitoring of the WAAM process. This new three-dimensional probe, based on the Ionic probe [75,146,147,148,149,150], was adapted to accommodate the weld bead curvature of the final layer. However, the results were less promising due to excessive noise caused by the inherent roughness of the part from the process [225]. Serrati et al. explored a different probe configuration that yielded better, though still not ideal, results. Their probe featured a “square” exciting coil designed to follow the semicircular geometry typical of the top of the WAAM part, and two “semicircular–triangular” sensing coils. These sensing coils were symmetrical but had their symmetry planes misaligned and oblique to the symmetry plane of the wall in the probe’s travel direction. The coils were wound in opposite directions to provide differential readings (Figure 13). This configuration improved the performance and a flexible PCB version of the probe was tested and validated [226].

### 3.8. ECT Probe Solutions for CFRPs

**Figure 14:**
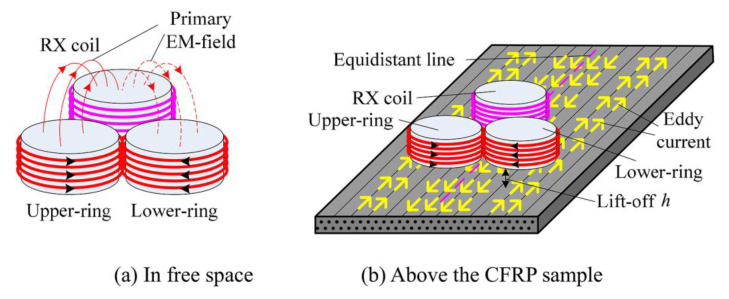
Principle of Lift-Off Insensitivity: The RX coil, designed in an 8-shaped configuration, has upper and lower rings that generate primary electromagnetic fields of equal strength but opposite directions along the equidistant line. This results in a total magnetic flux of zero penetrating the RX coil. Consequently, the RX coil’s output remains unaffected by the primary electromagnetic field produced by the TX coil [230].

Carbon fiber-reinforced polymers (CFRP) are increasingly used in lightweight applications such as aerospace and automotive manufacturing due to their high strength-to-weight ratio [227,228,229]. However, production defects like fiber misalignment, missing bundles, and wrinkles, as well as operational issues such as cracks, delamination, and impact damage, can lead to significant quality problems and increased costs [228,230]. Despite CFRP’s low electrical conductivity compared to metals, which makes conventional ECT challenging, the method still holds potential for defect inspection. CFRP’s conductive fibers and their inhomogeneous arrangement create a complex scenario for ECT compared to homogeneous metal samples. Addressing issues such as the appropriate test frequency, probe shape, and signal processing is crucial for the effective application of ECT to CFRP [231]. ECT has been employed to inspect various aspects of CFRP, including undulations in carbon fiber reinforcement fabrics, stacking sequence quality, fiber orientation, and curing effects [232,233,234]. It also helps characterize electrical properties across different orientations [235,236]. Given CFRP’s anisotropy and low conductivity, square coils can enhance electromagnetic coupling between the carbon fibers and coil wires. Wu et al. developed an ECT probe specifically for CFRP, featuring two planar square spiral coils, each with 5 mm sides printed on FR4, operating in transmission mode. The transmitter and receiver coils are aligned along the fiber orientation. In traditional transmission–reception (T-R) probes, eddy currents primarily develop in the probe’s middle area. However, due to CFRP’s strong anisotropy, the induced eddy currents in unidirectional laminates are stretched along the fiber orientation, making coils aligned with the fibers more sensitive. Therefore, square spiral coils were chosen over circular ones to better match the fiber alignment. When the transmitter and receiver coils are arranged perpendicular to the fiber orientation, the probe’s operation changes significantly. In this configuration, the coupling mainly depends on the middle area of the probe, which performs well in orthogonal directions, but less effectively for fibers in other orientations, though it can still be used to characterize fiber orientation [237]. Yin et al. designed multi-frequency eddy current sensors for a range of applications including bulk conductivity measurements, directionality characterization, and fault detection and imaging in CFRP samples [238,239]. They developed three sensors: the first was a circular air-cored coil for estimating bulk conductivity; the second, a ferrite-cored rectangular coil pair (one transmitter and one receiver); and the third, a smaller circular air-cored coil used for imaging damage sites. Both simple analytical and finite element (FE) models were used to describe the sensor responses, and these models showed good agreement with the experimental results [238,239]. Mizukami et al. focused on detecting fiber waviness in CFRPs by developing an eddy current (ET) probe specifically designed to detect both in-plane and out-of-plane waviness. The probe features three rectangular coils arranged in line: the first and third coils are identical and oriented perpendicular to the CFRP surface, while the center coil is oriented orthogonally to the other two. This setup allows the probe to perform three functions: detecting in-plane waviness, detecting out-of-plane waviness, and characterizing fiber orientations. The probe operates in different modes depending on the function being performed [240]. Additionally, the plane waviness size was studied using a probe with a vertical rectangular driver coil positioned above the waviness zone in the fiber direction, along with a pickup coil. The driver coil generates a magnetic field and induces eddy currents, while the pickup coil measures the resulting magnetic field generated by the drive current and the eddy currents [241]. Mizukami also proposed a method to select carbon fiber layers for inspection using a probe with rectangular driver and pickup coils oriented perpendicular to each other. By changing the in-plane azimuth of the probe, eddy currents can be concentrated in layers aligned with the fiber direction. This method was used to detect artificially induced in-plane waviness in cross-ply CFRP laminates [242]. Delamination detection has been explored using various techniques [243]. Notable methods include using artificial delamination created with interplay release film [244] and detecting extensive delamination during tension testing [245]. Mook et al. developed high-frequency eddy current sensors for the non-destructive characterization of CFRP. They designed two probes: a rotating probe with two bobbin coils operating in reflection mode and a static differential probe. The rotating probe can detect the fiber orientation without needing lateral movement, while the static probe is capable of visualizing the fiber orientation, local imperfections, resin-rich zones, delaminations, and impact damages [243]. Zhou et al. introduced a novel triple rectangular coil probe for delamination detection in CFRPs [246]. This probe comprises one horizontal rectangular coil for detection and two vertically aligned rectangular coils for excitation. The use of two excitation coils enhances eddy currents in the vertical direction within the sample, thereby improving the detection sensitivity. The spatial arrangement of the excitation and detection coils minimizes mutual inductance and reduces interference signals. The probe is designed to detect delaminations ranging from 10 mm to 30 mm in size and 0.05 mm to 0.15 mm in thickness. Simulations using COMSOL Multiphysics indicated that the probe could detect delaminations from both the top and bottom surfaces of a CFRP sample, including those deeper within the material. However, experimental tests have not yet been conducted [246]. Wu et al. addressed the challenges of signal interference and reduced sensitivity caused by variations in the probe-to-sample distance and random noise from lift-off changes. To mitigate these issues, they replaced the traditional circular transmitter (TX) coil in a T-R probe with an 8-shaped coil. This design ensures that the primary electromagnetic fields generated by the upper and lower rings of the 8-shaped coil are equal in strength but opposite in direction, as shown in Figure 14. Consequently, the total magnetic flux penetrating the receiver (RX) coil is zero, minimizing the impact of the primary EM field on the probe’s output. The probe operates within a frequency range of 10 MHz to 25 MHz and is suitable for in-plane waviness detection, defect identification, and characterizing fiber orientation in CFRPs [230]. Schmidt et al. investigated high-frequency ECT for quality assurance (QA) and process monitoring of CFRP parts produced by automatic fiber placement (AFP), a common production method in the aerospace industry [247,248]. They used a set of cured plates with various defects and uncured prepreg material to evaluate the effectiveness of EC testing in layup processes. Testing was conducted with three types of probes: a high-frequency absolute probe, a differential probe, and a transmission probe, all featuring helicoidal cylindrical coils. The differential probe was found to be unsuitable due to high noise levels, which complicated the image analysis. Both the transmission and absolute probes were capable of observing fiber orientations, with the transmission probe providing higher contrast and the absolute probe offering better resolution. Small defects as tiny as 6 × 6 mm could be detected at depths of several layers, with the transmission probe proving more effective for uncured prepreg and overlap detection due to its high contrast and resolution [247]. Advanced modeling approaches for eddy current propagation in CFRP showed good agreement with the experimental results [249,250,251,252].

**Figure 15:**
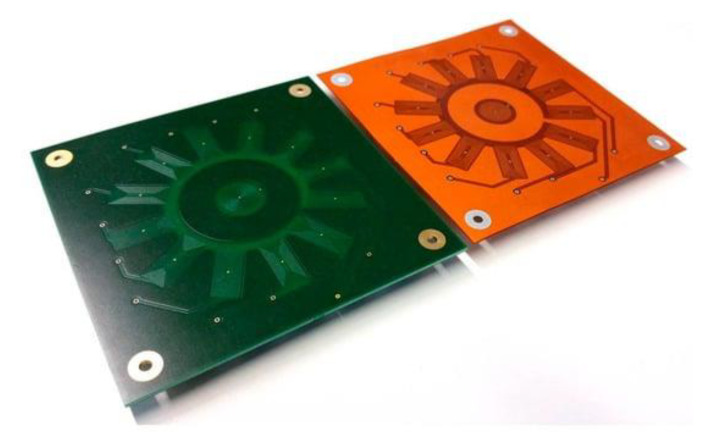
Rigid (**left**) and flexible (**right**) PCB-based eddy current sensor array [254].

Zhang et al. developed a flexible ECT probe with a front-end differential setup for inspecting CFRP samples with curved surfaces. This probe operates at very high excitation frequencies and is designed to handle irregularly shaped structures [253]. The probe consists of two spiral coils fabricated on a flexible printed circuit board (FPCB), which can conform to the surface of the test sample. Each coil has 16 turns distributed across four layers of the FPCB. The prototype probe was tested on CFRP plates and tubes with machined defects, as well as curved CFRP samples with both machined and naturally occurring impact damages. It successfully detected small defects (2 mm length, 1 mm width, and 0.4 mm depth) on curved CFRP surfaces, demonstrating high sensitivity. The study also discussed the effect of the excitation frequency, recommending a medium frequency of around 20 MHz, and included a 3D FEM model to analyze the eddy current distribution and the impact of CFRP’s anisotropic electrical conductivity [253]. Berger et al. introduced a sensor concept for detecting textile defects during the preforming of semi-finished carbon fiber parts. They developed a reflection probe using printed circuit boards (PCBs), which features one emitting coil with a circular cross-section (12 mm diameter and 12 turns, as seen in Figure 15) and 12 pickup coils with rectangular shapes (8 mm × 12 mm, 10 turns each) [245]. This approach aims to create static eddy current arrays that can be integrated into the preforming stage of Resin Transfer Molding. The implementation of this technology significantly reduces the measurement times during the quality inspection of carbon fiber preforms. The results demonstrated that fiber tow orientations could be determined with high reliability using this system [254].

Unidirectional carbon fiber-reinforced polymer composites (UD CFRP) are high-performance materials for structural components; however, they exhibit low damage tolerance [255,256]. Condition monitoring is, therefore, required in safety-critical applications. Machado et al. developed a customized EC system capable of detecting fiber breaks [257,258] and delaminations [54,259] in UD CFRP. Their system could perform inspections at high speeds (4 m/s). For detecting fiber breaks, they used a probe featuring two 45° parallelogram spiral coils operating in differential bridge mode with a 3 mm lift-off. The PCB configuration was chosen to maximize the proximity of the winding to the CFRP surface, enhancing the sensitivity due to a closer interaction with the eddy current changes. The 45° parallelogram spiral coils demonstrated an excellent performance, successfully detecting lateral cuts of a 0.2 mm width in CFRP with a clear signal [258].

**Figure 16:**
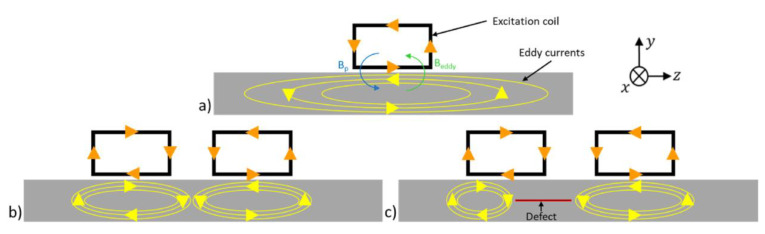
Representation of the eddy currents flow: (**a**) one coil where the high anisotropic effect can be seen in the EC flow; (**b**) two coils excited with opposite current direction; and (**c**) two coils excited with opposite current direction when a delamination-type defect is present and its consequence to the EC flow [54].

For delamination detection, the challenge arises from the highly anisotropic nature of the material, making inspection and probe design particularly difficult. Machado et al. employed two rectangular coils operating in bridge differential mode, oriented vertically. To control the spread of the EC along the length direction, the coils were configured to induce currents in both clockwise and counterclockwise directions, as illustrated in Figure 16. Under normal conditions, without defects, the EC flows symmetrically within the coils’ plane, resulting in a balanced magnetic field and equal impedance in both coils. However, when a delamination defect is present, it disrupts the vertical EC flow at the ends of the coils, leading to changes in the probe’s response. Multilayered PCBs were used to achieve the required coil turns, with an innovative vertical disposition allowing for multiple windings. Each PCB, 1.55 mm-thick, consisted of eight layers, enabling each coil to have the desired 40 windings. This design proved effective in detecting both horizontal and vertical delaminations and showed sensitivity to most fiber breaks [54].

In the context of aluminum honeycomb sandwich structures with CFRP panels, Ren et al. conducted an experimental study using ECT. The structure, known for its high specific strength, stiffness, heat insulation, and anti-fatigue properties, is commonly used in aerospace, shipbuilding, and automotive industries [260,261,262]. Ren et al. designed an EC probe with two 6 mm-diameter pancake coils, wound around a cylindrical ferrite core, one positioned above and one below the core. The probe operates in a bridge differential mode with each coil having 100 turns and shielded by a copper cover. The amplitude of the differential voltage was measured at various positions during scanning. The results demonstrated that the probe was effective in detecting core defects and impact damages within the sandwich structure [57].

### 3.9. ECT Probe Solutions for GFRPs

ECT can be adapted for inspecting non-conductive materials, such as Glass Fiber Reinforced Polymers (GFRP), which are valued in industries like aerospace and automotive for their strength and corrosion resistance [263,264,265]. One adaptation involves permittivity sensing, which differs from traditional ECT by focusing on how a material responds to an electric field rather than its electrical conductivity. Permittivity sensing measures how much resistance a material offers to the formation of an electric field within it. In GFRP inspection, this technique involves modifying the ECT setup to detect changes in the permittivity caused by defects such as delaminations, voids, or variations in the material composition. Defects alter the permittivity compared to the surrounding intact material, which can be detected by capacitive sensors or electrodes integrated into the ECT probe. As the probe scans the GFRP surface, it measures the electric field and analyzes deviations to identify potential defects. The key benefits of permittivity sensing include its non-contact nature, which preserves the material’s integrity, and its capability to detect subsurface defects without needing direct access to both sides of the material. It is particularly useful for complex shapes and structures where traditional methods might be impractical. However, implementing permittivity sensing presents challenges. Precise calibration is required to account for variations in the material composition, thickness, and environmental conditions. Factors such as temperature and humidity can affect the permittivity measurements, necessitating careful control and compensation to ensure accurate defect detection [266].

## 4. ECT Simulation for Probe Design

Numerical simulations play a pivotal role in enhancing the effectiveness and understanding of eddy current testing (ECT), particularly in the design of ECT probes. By allowing the detailed analysis of electromagnetic fields and their interactions with conductive materials, the simulations provide crucial insights that complement the experimental methods. Central to these simulations are Maxwell’s equations, which govern electromagnetic phenomena. Using techniques like the Finite Element Method (FEM), researchers can model eddy currents (EC) and the resulting magnetic fields in response to varying probe configurations and material properties. One of the primary advantages of a numerical simulation is its ability to model various defect types—such as cracks, voids, and inclusions—within the test material [267]. By altering the geometry, orientation, and size of these defects, researchers can predict their impact on the eddy current response. This predictive capability is essential for optimizing the design of ECT probes, as it helps identify the most effective configurations and operational parameters, such as the probe geometry and excitation frequency, to enhance defect detection. In addition to optimizing designs, numerical simulations also provide a means for the calibration and validation of experimental setups. By comparing the simulation results with the actual experimental data, engineers can refine their models, ensuring that the numerical approach accurately reflects real-world conditions. This iterative process bolsters confidence in both the simulation outcomes and the efficacy of the ECT system. Furthermore, simulations can be integrated with other NDT methods, such as ultrasonic testing or thermography, creating a hybrid approach that allows for the comprehensive evaluation of components and leverages the strengths of each technique to enhance the overall defect detection capabilities [268,269,270]. Various software packages, including ANSYS [48,55,56,183], COMSOL Multiphysics [140,166], and CST Studio Suite [182,271], offer robust platforms for simulating ECT. These tools often feature built-in libraries for material properties and defect models, simplifying the modeling process and enabling the rapid prototyping of different ECT configurations. As the computational power continues to grow and algorithms advance, the fidelity of numerical simulations in ECT is poised for improvement. Future developments may include real-time simulations that adapt to changing conditions during inspections and enhanced machine learning techniques that analyze simulation data to further optimize inspection strategies. Through these innovations, numerical simulations stand to significantly enhance the precision and applicability of eddy current testing across various industries. With the advent of artificial intelligence (AI) and deep learning, the field of ECT simulation is experiencing a paradigm shift. AI algorithms, particularly those based on deep learning, can analyze vast amounts of simulation data to identify patterns and optimize probe designs in ways that were previously unimaginable [272,273]. These advanced techniques can enhance the predictive capabilities of simulations, allowing for real-time adaptations during inspections and improved accuracy in defect detection [274]. Machine learning models can also be trained to recognize subtle variations in eddy current responses that might indicate early-stage defects, thus enhancing the sensitivity and reliability of ECT systems [275,276]. As computational power continues to grow and algorithms advance, the fidelity of numerical simulations in ECT is poised for significant improvement. Future developments may include real-time simulations that adapt to changing conditions during inspections and enhanced machine learning techniques that analyze simulation data to further optimize inspection strategies. Through these innovations, numerical simulations stand to significantly enhance the precision and applicability of eddy current testing across various industries [277].

In summary, numerical simulations significantly aid in the design and optimization of ECT probes, providing valuable insights that improve defect detection capabilities. By facilitating the comparison between the simulation and experimental results, researchers can refine their approaches, ensuring that the ECT systems are both accurate and effective in real-world applications. The integration of AI and deep learning into this process promises even greater advancements, paving the way for more intelligent and adaptive ECT solutions.

## 5. Conclusions

This work has highlighted significant advancements and ongoing challenges in the field of eddy current testing (ECT) probe design for non-destructive testing (NDT) applications. The integration of numerical simulations, primarily through techniques like the Finite Element Method (FEM), has revolutionized the design process. These simulations enable a detailed analysis of electromagnetic interactions, providing invaluable insights into optimizing probe configurations for enhanced defect detection. The ability to model various defect types and predict their impact on the eddy current response has proven crucial for refining probe designs and operational parameters. By comparing simulated results with the experimental data, researchers can iteratively improve their models, ensuring accuracy and effectiveness in real-world applications.

Several innovative probe designs have been developed to address specific challenges in ECT, including detecting small defects in complex geometries and improving real-time monitoring capabilities. Advances in material properties, sensor configurations, and signal processing have all contributed to the improved performance of ECT probes. Despite these advancements, challenges remain, particularly in achieving precise calibration to account for variations in the material composition and environmental conditions.

Future research directions include enhancing the fidelity of numerical simulations and exploring real-time adaptive simulations that respond to changing inspection conditions. Additionally, the application of machine learning techniques to analyze simulation data and optimize inspection strategies holds significant potential. Continued innovation in ECT probe design and simulation will undoubtedly enhance the precision and applicability of eddy current testing across various industries.

In addition to these simulation-focused advancements, there are several non-simulation-related research avenues worth exploring. One area is the development of high-temperature ECT probes that can maintain sensitivity and reliability under extreme conditions, such as in power plants or aerospace applications, where standard probes often fail. Another promising direction is the design of hybrid ECT probes that integrate multiple sensing techniques (e.g., combining eddy current with ultrasonic or infrared sensors) to enable comprehensive inspections in complex environments. Furthermore, research could focus on the improvement of ECT probes for multi-material interfaces, addressing the challenges of inspecting joints and interfaces between different materials, such as metal–composite or metal–ceramic, to provide accurate readings across diverse material types.

In summary, the advancements in ECT probe design and numerical simulations have significantly improved the capabilities of non-destructive testing, making it more accurate and reliable. However, ongoing research and development are essential to address existing challenges and fully realize the potential of these technologies in diverse industrial applications.

## Data Availability

No new data were created or analyzed in this study. Data sharing is not applicable to this article.
